# Successful reproduction of a large EEG study across software packages

**DOI:** 10.1016/j.ynirp.2023.100169

**Published:** 2023-05-27

**Authors:** Aya Kabbara, Nina Forde, Camille Maumet, Mahmoud Hassan

**Affiliations:** a LASeR - Lebanese Association for Scientific Research, Tripoli, Lebanon; b MINDig, F-35000, Rennes, France; c Inria, Univ Rennes, CNRS, Inserm, IRISA UMR 6074, Empenn ERL U 1228, Rennes, France; d School of Science and Engineering, Reykjavik University, Reykjavik, Iceland

**Keywords:** Electroencephalography, Reproducibility, EEG preprocessing, Inter-software variability

## Abstract

As an active field of research and with the development of state-of-the-art algorithms to analyze EEG datasets, the parametrization of Electroencephalography (EEG) analysis workflows has become increasingly flexible and complex, with a great variety of methodological options and tools to be selected at each step. This high analytical flexibility can be problematic as it can yield to variability in research outcomes. Therefore, growing attention has been recently paid to understand the potential impact of different methodological decisions on the reproducibility of results.

In this paper, we aim to examine how sensitive the results of EEG analyses are to variations in preprocessing with different software tools. We reanalyzed the shared EEG data (N = 500) from (Williams et al., 2021) using three of the most commonly used open-source Matlab-based EEG software tools: EEGLAB, Brainstorm and FieldTrip. After reproducing the same original preprocessing workflow in each software, the resulting event-related potentials (ERPs) were qualitatively and quantitatively compared in order to examine the degree of consistency/discrepancy between software packages. Our findings show a good degree of convergence in terms of the general profile of ERP waveforms, peak latencies and effect size estimates related to specific signal features. However, considerable variability was also observed in the magnitude of the absolute voltage observed with each software package as reflected by the similarity values and observed statistical differences at particular channels and time instants. In conclusion, we believe that this study provides valuable clues to better understand the impact of the software tool on the analysis of EEG results.

## 1 Introduction

Electroencephalography (EEG) is a well-established technique for measuring the electrical fluctuations generated by the brain at high temporal resolution. Due to its non-invasiveness, low cost and ease-of-use EEG has been gaining increasing interest in uncovering the functional brain activity underlying various brain conditions including disorders, emotions, information processing and resting state (Lopes da Silva, 2013; Urigüen and Garcia-Zapirain, 2015).

Typically, the EEG electrodes capture a mixture of neural activity and non-neural-related artifacts which can be physiological (e.g. eye movements or muscle contractions) or external to the human body (e.g. power line or interference with other electrical devices) (Urigüen and Garcia-Zapirain, 2015). Thus, to study the EEG signal, it is of great importance to first carefully reduce the influence of contaminating artifacts while preserving the neural activity. This is the aim of the preprocessing stage which is carried out so as to derive clean EEG signals suitable for further statistical analysis. Preprocessing typically includes multiple steps, such as line noise removal, re-referencing, artifact rejection, filtering, epoch selection, bad channels detection and interpolation. Although there is a general agreement in the scientific community on the main steps that should be considered in the preprocessing pipeline, each step can be approached through many algorithmic strategies with different sets of assumptions, and parameter choices (Boudewyn et al., 2018; Croft et al., 2005; Croft et al., 2005; Šoškić et al., 2021, 2022). Thus, the preprocessed signals are the result of multiple individual and user-dependent decisions, made over a potentially long and ordered pipeline. More specifically, the chain of decisions is not only limited to adjusting the features incorporated in each preprocessing step, but may even start before the preprocessing is performed - i.e, when selecting the adequate software tool.

In this context, many efforts have focused on proposing guidelines for researchers to choose between the existing cleaning methods depending on the application and user's requirements. Among these efforts (Islam et al., 2016; Ranjan et al., 2021), present extensive reviews of the existing state-of-the-art artifact cleaning methods by showing the pros, cons and suitability in particular applications.

Recently, growing attention has been paid to evaluate the variability and comparability of results obtained with different preprocessing methods and parameter choices (Barban et al., 2021; Clayson et al., 2021; Robbins et al., 2020). The main objective of these studies was to test how much the variability in cleaning methods can impact the conclusions of a study. For instance, the effect of three artifact removal algorithms (ICA-LARA, ICA-MARA and Artifact Subspace reconstruction (ASR)) on EEG characteristics and event-related measures was analyzed and compared across 17 EEG studies (Robbins et al., 2020). Results highlight the existence of significant differences between results particularly after eye blinks artifacts have been removed. Others were interested in testing the ability of different blind source separation methods to remove synthetic/modeled noise sources corrupting real EEG signals (Barban et al., 2021). The main results show that there is no method that can be considered as an all-purpose algorithm, and the choice of the adopted method should be driven by the specific needs of users (such as the computational capacities, or the temporal constraints). Trying to optimize the preprocessing pipeline for the event-related potentials (ERPs) (Clayson et al., 2021; Šoškić et al., 2022), examined the impact of many possible methodological choices on the data quality and the experimental effects through data multiverse analysis. Both studies highlighted the substantial impact of several parameters such as the filter cut-off, artifact detection method, baseline adjustment, reference, scoring electrodes and others on the study's outcomes.

While the above studies provide important insights on the effect of either the preprocessing stages, the preprocessing algorithms or the parameter choices, the preprocessing of the signals were carried out using a single software tool. Yet, there are many tools available to study the EEG signal including open-source and commercial software packages. Among the open-source packages, EEGLAB (Delorme and Makeig, 2004), Brainstorm (Tadel et al., 2011), MNE (Gramfort et al., 2014), FieldTrip (Oostenveld et al., 2011) and Automagic (Pedroni et al., 2019) are the most commonly used. Each toolbox has its own way to organize and format the data, to implement functions and to define their arguments, parameters, optimal and default values. Another important difference between tools resides in the availability of the desired preprocessing steps as well as the parameters that can be accessed for each step.

Here we investigate how sensitive the results of EEG analyses are to variations in software packages when using the same dataset and aligned preprocessing methods. To this aim, we reanalyzed data (N = 500) from a recent study by Williams and colleagues (Williams et al., 2021) and reproduced the study using three software packages to quantify the observed differences in the final results. Our objective was first to reproduce the main figures of (Williams et al., 2021) by replicating the original preprocessing pipeline used within each software tool. We compare three of the most commonly used open-source Matlab toolboxes: Brainstorm (first release in 2000, 2559 citations as of 11/10/2022 according to Google Scholar), EEGLAB (first release in 2004, 18373 citations) and FieldTrip (first release in 1999, 7448 citations) in order to achieve two main objectives: 1) Study whether the main findings of the original paper – including ERP waveform as well as effect size estimates related to selected ERP features – could be reproduced within each software package. 2) Quantify variations observed across software packages.

## 2 Materials and methods

### 2.1 Material

#### 2.1.1 Dataset

We used the dataset previously analyzed in (Williams et al., 2021), and publicly available at www.osf.io/65x4v/. In brief, this dataset comprises data from 500 undergraduate healthy students (341 females, 154 males, mean age = 21.71 years old, 440 right handed, 53 left handed) recruited by the University of Victoria. These participants were selected amongst a total of 637 subjects as they had provided signals with a high data quality. The study was approved by the University of Victoria's Human Research Ethics Board and all participants provided written informed consent before any data acquisition.

We chose to reproduce this study by Williams and colleagues for two main reasons. First the availability of the raw data and of the preprocessing and analysis scripts made it possible for us to recompute the original results to serve as a reference for our subsequent analyses. Second, the large number of participants (N = 500) made this study less sensitive to a lack of reproducibility that would be due to a small sample size (Button et al., 2013; Ioannidis, 2005).

#### 2.1.2 Experimental protocol

**Fig. 1:**
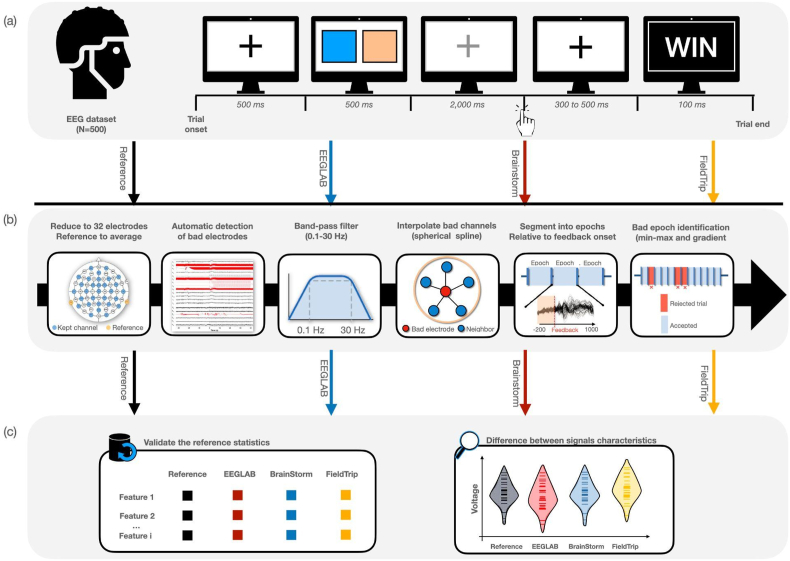
Overview of the study. (a) We used shared EEG data from (Williams et al., 2021) with 500 participants performing a simple gambling task of six blocks composed of 20 trials. (b) This dataset was then preprocessed using the different software tools: Reference (using the code published with the original paper), EEGLAB, Brainstorm and FieldTrip. The preprocessing steps to be performed in each tool included: reduction to 32 electrodes, reference to average, automatic detection of bad electrodes, band-pass filtering (0.1–30 Hz), interpolation of bad channels, segmentation into time-locked epochs (from −500 to 1300 ms around the feedback stimulus) and removal of artifactual trials (identified with 10 μV/ms gradient and 100 μV maximum–minimum criteria). (c) The preprocessed signals derived from the four preprocessing codes were used to reproduce the reference statistics and validate the hypotheses. A quantitative comparison between the resulting signals was also conducted in terms of signal features (peak latency, mean peak, maximum peak and base-to peak features - please refer to materials and methods section for more details). Image credits: EEG cap CC-BY Wikimedia Commons by CIV The Noun Project.

Participants completed a simple gambling task following a two-armed bandit task. This task was chosen by Williams and colleagues in (Williams et al., 2021), as it is the most commonly used paradigm to evoke the reward positivity ERP which was the subject of investigation of the reference paper (Proudfit, 2015). The pipeline of our study is summarized in Fig. 1.

The acquisition session consisted of six blocks of 20 trials (see Fig. 1a). Each trial was initiated by a black fixation cross displayed for 500 ms, followed by a 500 ms display of two colored squares. Then, the fixation cross turned gray to prompt the participant to select one of the two colored squares (left or right) within a 2000 ms time limit. After that, a black fixation cross was presented for 300–500 ms, and a simple feedback (“WIN” for gain, “LOSE” for loss) was shown for 1000 ms. The final objective of this task for the participant was to win as often as possible. This was possible for the participant by determining (while computing the task) which square would bring the most successful rate (60% for one square vs. 10% for the other one). The same pair of colors was used for all the trials of the same block, and the squares locations were randomized for each trial.

#### 2.1.3 Data collection

EEG data were acquired from either 64 or 32 electrode (Ag/AgCl) EEG systems (ActiCAP, Brain Products, GmbH, Munich, Germany) using Brain Vision Recorder. Data were originally sampled at 500 Hz and low-pass filtered below 245 Hz. During the recording, all electrodes impedance were kept under 20 kΩ in all participants.

### 2.2 Methods

#### 2.2.1 Original preprocessing pipeline

The preprocessing pipeline adapted by the reference paper was performed in Matlab using scripts available at www.osf.io/65x4v/(the main file is named *‘RewardProcessing_Preprocessing.m’*), where some functions have EEGLAB dependencies. Briefly, the pipeline consists of processing data twice wherein the first pass was used to identify noisy or damaged electrodes, and the second pass was done to process data. The steps (Fig. 1B) are performed as follows:

• **Reduce the number of electrodes to 32 electrodes** (for all data that were collected with a 64 electrode EEG system).
• **First processing pass - Detect artifactual channels:** Practically, the detection of artifactual channels can be approached in different ways. Among these strategies, Williams and colleagues chose to mark as ‘bad’, the channels that provided a high trial rejection rate. Data were first re-referenced to a linked mastoid reference (using TP9 and TP10 electrodes) and band pass-filtered between 0.1 and 30 Hz (Butterworth, order 4). A notch filter at 60 Hz was also applied. Afterwards, authors have corrected eye blinks after manually identifying the corresponding independent components (ICs) reflective of blinks. Time-locked epochs around the feedback stimulus onset (from −500 to 1500 ms) were then extracted, and baseline corrected using a −200 to 0 ms window. An artifactual trial (i.e epoch) was identified with 10 μV/ms gradient and 100 μV maximum–minimum criteria. Ultimately, an electrode was considered noisy or artifactual if it exceeded a trial rejection rate of 40%. The goal of this first pass was to detect the artifactual channels to be interpolated. Hence, no changes were effectively applied on the underlying signals. Thus, many of the processing procedures performed in this step (including re-referencing, filtering, .etc) were then replicated, and applied to the original signal during the second processing pass.

**Second processing pass:**

• **Re-reference data to linked mastoids** (using TP9 and TP10 electrodes)
• **Apply a band pass-filter** between 0.1 and 30 Hz (Butterworth, order 4) and a **notch filter** at 60 Hz.
• **Interpolate the detected artifactual channels** using the spherical spline method.
• **Detect and remove the eye blinks artifacts** using independent component analysis (ICA) after manually selecting the blinks components via topographic maps and component loadings. This step was removed from the pipeline used in the current manuscript as it contained manual processings.
• **Extract the time-locked events** using a segment window of −500 to 1300 ms relative to the feedback stimulus.
• **Baseline correction** by removing from each channel the average of the values computed over the baseline (−200 to 0 ms).
• **Reject trials** that exceed a gradient of 10 μV/ms and a maximum-minimum voltage of 100 μV.
• **Compute the ERPs of gain and loss conditions** by averaging the corresponding epochs.ERPs were trimmed to −200 to 1000 ms. Authors were also interested in analyzing the grand averaged ERP, denoted the reward positivity, obtained as the result of subtraction between the gain condition and the loss condition.

In the original preprocessing pipeline proposed by the authors, a manual procedure – i.e., a human-based and visually guided procedure – was used to detect the components corresponding to the eye blinking noise. However, this step is not only time-consuming to be carried out in each software tool for 500 subjects, but more importantly also introduces inter-rater variability as it is open to the level of expertise and variability across different raters performing the manual detection. In order to focus on inter-software variability only, this step was removed from the preprocessing pipeline. In addition, some particular channels were detected as bad via visual inspection. This step was also removed in the current manuscript when we reproduced the results using the original script.

#### 2.2.2 Comparison across toolboxes

We selected three of the most widely used software packages available to preprocess EEGs and reproduced the reference preprocessing pipeline in each.

All code to reproduce the preprocessing pipelines is available at: https://github.com/Inria-Empenn/EEG_preprocessing (released on Zenodo, doi: 10.5281/zenodo.6918329) and more details are provided below on the algorithms and parameters chosen in each software package.

##### 2.2.2.1 EEGLAB

The EEGLAB preprocessing script was assembled and run for all the 500 subjects as follows:

• Load the data using *pop_loadbv.m*
• Reduce data into 32 channels using *pop_select.m*
• Automatically detect the noisy channels with the substeps detailed in *‘First processing pass - Detect artifactual channels’* of section 2.2.1 using EEGLAB functions**.** As these substeps were replicated in the second processing pass (as pointed in section 2.2.1), the names of the used functions as well as the parameters selected are listed in the following.
• Re-reference the signals to linked mastoid electrodes as performed in *‘Second preprocessing pass - Re-reference data to linked mastoids’* using *prop_reref.m.*
• Filter the signals between 0.1 and 30 Hz using *pop_eegfiltnew.m* as performed in *‘Second preprocessing pass - Apply a band-pass filter’.* This function uses a hamming window-based finite impulse response (FIR) filter with an order of 16500, determined as an optimal filter order following the equation: 3.3/(df/sampling rate), where df is the lowest pass-band edge equal to 0.1.
• Interpolate the detected noisy channels as performed in *‘Second preprocessing pass - Interpolate the detected artifactual channels’,* using the spherical spline method *pop_interp.m*
• Divide the signals into time-locked epochs as performed in *‘Second preprocessing pass - Extract the time-locked events’,* using the function *pop_epoch.m*, and apply baseline correction as in ‘*Second preprocessing pass - Baseline correction’,* using *pop_rmbase.m* function
• Reject the artifactual trials using the *pop_eegthresh.m* function for which the lower and upper amplitude limits can be identified by the user as in *‘Second preprocessing pass - Reject trials’.* Here, we set the lower limit to −50μV and the upper limit to 50 μV, in a way to follow the same parameters of the trial rejection procedure as adopted in the reference paper.
• Compute the ERPs (for gain and loss conditions) as in *‘Second preprocessing pass - Compute the ERPs of gain and loss conditions’***,** as well as the grand averaged ERP

The analysis was conducted using EEGLAB v2022.1 (RRID: SCR_007292).

##### 2.2.2.2 Brainstorm

The list of steps used to perform the preprocessing in Brainstorm are as follows:

• Detect the noisy channels with the substeps detailed in *‘First processing pass - Detect artifactual channels’* of section 2.2.1 using Brainstorm functions**.** As these substeps were replicated in the second processing pass (as pointed in section 2.2.1), the names of the used functions as well as the parameters selected are listed in the following.
• Re-reference to linked mastoids (using TP9 and TP10) as performed in *‘Second preprocessing pass - Re-reference data to linked mastoids’* using *process_eegref*
• Apply the notch filter at 60 Hz using *process_notch* as performed in *‘Second preprocessing pass - Apply notch filter’.*
• Apply a band-pass filter between 0.1 and 30 Hz as performed in *‘Second preprocessing pass - Apply a band-pass filter’,* with a linear phase FIR filter using the process *process_bandpass.* The filter order calculated was 18128 determined by the means of the Kaiser method.
• Interpolate the detected noisy channels as performed in *‘Second preprocessing pass - Interpolate the detected artifactual channels’,* using an interpolation of the neighbors weighted by distance method with *p*rocess_eeg_interpbad.
• Segment data into time-locked epochs as performed in *‘Second preprocessing pass - Extract the time-locked events’,* using *process_import_data_event.*
• Apply baseline correction as in ‘*Second preprocessing pass - Baseline correction’,* using the baseline period from −200 ms to 0 using *process_baseline.*
• Detect and reject the bad trials using a peak to peak of 100 μV using *process_detectbad,* as in *‘Second preprocessing pass - Reject trials’.*
• Compute the ERPs as in *‘Second preprocessing pass - Compute the ERPs of gain and loss conditions’***,** using *process_average*

The analysis was conducted using brainstorm version 22.07.29 (RRID: SCR_001761).

##### 2.2.2.3 FieldTrip

FieldTrip toolbox is not a software with a user interface, but rather a collection of functions. Thus, a Matlab script, in which a sequence of FieldTrip functions are called, is considered as an analysis protocol in FieldTrip. Each of the functions of the toolbox takes as input the data that was produced by the previous function. To allow a function to implement a specific algorithm, particular parameters can be specified via a configuration structure *cfg*. Here, we used the major functions ft_preprocessing, *ft_artifact_clip*, *ft_channelrepair*, *ft_redefinetrial, ft_rejectartifact* and ft_timelockanalysis.

More precisely, the FieldTrip preprocessing script was assembled and run for all the 500 subjects as follows:

• Bad channels were detected with the substeps detailed in *‘First processing pass - Detect artifactual channels’* of section 2.2.1 using FieldTrip functions**.** As these substeps were replicated in the second processing pass (as pointed in section 2.2.1), the names of the used functions as well as the parameters selected are listed in the following.
• Data were reduced to 32 channels, re-referenced to linked mastoids as performed in *‘Second preprocessing pass - Re-reference data to linked mastoids’*, filtered by a Butterworth filter (order = 4) as performed in *‘Second preprocessing pass - Apply band-pass filter’* using *ft_preprocessing* with *cfg.channel*, *cfg.refchannel*, *cfg.bpfreq*, *cfg.bpfilttype*, *cfg.bpfiltord* being adequately defined.
• The interpolation of the detected bad channels was done using *ft_channelrepair*, as performed in *‘Second preprocessing pass - Interpolate the detected artifactual channels’,* with *cfg.badchannel* being identified.
• The segmentation into time-locked epochs to win and loss conditions as performed in *‘Second preprocessing pass - Extract the time-locked events’,* was done using *ft_redefinetrial* where *cfg.trialdef* is configured.
• Baseline correction was performed as in ‘*Second preprocessing pass - Baseline correction’* using *ft_preprocessing* after defining *cfg.baselinewindow.*
• A trial is detected as bad, as in *‘Second preprocessing pass - Reject trials’,* using *ft_artifact_threshold* if it exceeds a min-max voltage of 100 μV following the same criteria of the reference paper, then rejected using *ft_rejectartifact.*

The ERPs were computed using ft_timelockanalysis as in *‘Second preprocessing pass - Compute the ERPs of gain and loss conditions*.

The analysis was conducted using FieldTrip version 20220104 (RRID: SCR_004849).

#### 2.2.3 Modified preprocessing pipelines

The channel detection procedure used in the reference paper was not originally available in any of the tested tools (EEGLAB, Brainstorm, FieldTrip) and involved preprocessing the data twice which is atypical in EEG analyses. Thus, in addition to the original pipeline, we computed two alternative pipelines to use more widespread approaches of the channel detection procedure in EEGLAB, Brainstorm and FieldTrip:

We modified the channel detection step to use each tool's preferred method to automatically detect bad channels. In practice, EEGLAB incorporates different methods, such as ‘clean_rawdata’ and the PREP pipeline (Bigdely-Shamlo et al., 2015) that automatically detect bad channels based on signal characteristics in terms of spatial (correlation with neighbors), spectral (such as frequency noise.) or time features (such as amplitude deviation). However, Brainstorm and FieldTrip do not provide an advanced automatic approach to detect the noisy channels. Instead, users of these two toolboxes could automatically detect the channels showing flat signals. To avoid unfair comparisons, we chose to limit our search to the automatic detection of flat channels. For EEGLAB, *‘clean_rawdata’* was used to detect channels with no signal variation for a duration of longer than a specific time window length (default 5s). For Brainstorm, ‘*process_detectbad’* was used. For FieldTrip, *ft_artifact_clip* with *cfg.artfctdef.clip.timethreshold* was used to detect channels showing signals being completely flat for a given time window (which was set to 5s). All the other steps with their corresponding parameters mentioned in the previous sections remained untouched.

We modified the channel detection step to use each tool's preferred method to automatically detect flat channels. But, here, the peak-to-peak threshold used to detect bad trials was increased to 200 μV (instead of 100 μV).

#### 2.2.4 Reproduction of the ERP analysis

In (Williams et al., 2021), the authors focused on analyzing the neural feedback processing based on multiple measures of reward positivity. Many of these measures rely on the ERP, which attempts to characterize the neural activity by examining the peaks and troughs of the averaged signals time-locked to events of interest (Picton et al., 1995). More specifically, the authors have first computed the ERPs for each condition (gain and loss) within each participant. Difference ERPs were also extracted by subtracting the ERP related to the loss condition from that related to the gain condition. Then, four quantitative ERP-based features were determined corresponding to FCz electrode, the most commonly used electrode in the context of reward positivity (Sambrook and Goslin, 2015). *Peak time of the reward positivity*: computed for each participant by finding the peak amplitude of the difference ERP waveform. *Mean peak*: Average of the voltages ±46 ms surrounding the peak location. *Maximum peak*: Largest amplitude within the 200–400 ms time window. *Base-to-peak*: Measure computed by subtracting the minimum voltage of the trough immediately prior to the reward positivity from the maximum peak measure.

The mean, maximum and base-to-peak metrics were computed for the gain and loss ERPs and the difference ERPs of each participant. In our study, we followed the same ERP exploration and features extraction procedures after obtaining the preprocessed signals from the different software tools.

### 2.3 Comparison methods

We applied three separate quantitative methods to measure the discrepancy between the results obtained within each software. First, the statistical comparisons among metrics (mean, maximum, base-to-peak and peak-time) obtained by the different software tools were performed using Wilcoxon ranksum test. For each metric of interest, we compared the values obtained by the reference, EEGLAB, Brainstorm and FieldTrip for all participants. These comparisons provide a quantification of the level of (dis)agreement between each pair of software tools about the ERP features of interest. The statistical significance level was set to *p* < 0.01 and Bonferroni correction was used to address the multiple comparisons issue across the number of tests performed (6 comparisons).

Second, we evaluated the variability of results by computing the similarity between the ERPs obtained by the different software tools, when considering all the EEG channels. In fact, in their paper, Williams and colleagues have only considered the FCz electrode as it was shown to be the electrode that extracts the most relevant information related to their topic of interest (i.e the reward positivity). Here, however, we are also interested in studying the effect of the software tool on the preprocessed EEG signals of all the recording channels. Thus, for each participant, we assessed the similarity between two software tools *S1* and *S2* using Pearson's correlation measure as follows:

$$Sim_{p}\left(S1,S2\right)=\frac{1}{C}\times \sum_{c=1}^{C}r\left({ERP}_{p,c}^{S1},{ERP}_{p,c}^{S2}\right)$$

Where *p* is the considered participant, *C* is the number of channels. ${ERP}_{p,c}^{S1}$ and ${ERP}_{p,c}^{S2}$ denote the ERP signal obtained at channel c from the software S1 and the software S2, respectively. Pearson's measure was used as variables were checked to be normally distributed using Kolmogorov-Smirnov test and no outlier was detected. More precisely, Kolmogorov-Smirnov test indicates that the ERP signals for the 32 channels follow a normal distribution with D(500) ranging from 0.02 to 0.1 and a p-value ranging from 0.1 to 0.4 (greater than 0.05).

In addition, we were interested in precisely describing the ERP differences between software tools in terms of temporal and spatial characteristics. This was done by assessing the statistical difference between ERP distributions at each time sample and each channel using cluster-based permutation tests (Maris and Oostenveld, 2007). Multiple comparisons (across 32 electrodes and 600 time samples) were effectively accounted for by this method. To compare ERPs obtained from two different tools, t-statistics quantifying the EEG amplitude differences were computed and all the corresponding electrode/sample combinations having a p-value lower than 0.05 were identified. Among the identified electrodes and samples, the adjacent ones are clustered and the sum of t-values within each cluster was calculated. Afterwards, the method generates multiple random partitions by changing the assignments of trials between tools. After calculating the t-statistics on each random partition, we obtain a null distribution of the summed cluster values. Finally, p-values were calculated as the proportion of random partitions showing a t-value larger than the observed one.

In addition, we compared the ERP generated by the different tools in terms of the data quality. This was assessed in terms of the Standardized Measurement Error (SME). As reported in (Luck et al., 2021), the SME is the standard error of measurement for a particular score. Here, the score we chose was the mean peak score (average of the voltages ±46 ms surrounding the peak location) as this score is commonly used in the context of reward positivity (Sambrook and Goslin, 2015). For each subject, we used bootstrapping to compute the SME for the mean peak score of both gain and loss ERP generated by the different software tools. The bootstrapping procedure consists of 1000 iterations in which new averaged ERP waveforms were created each time for the gain and loss trials from a randomly selected set of trials. This provides 1000 mean peak scores for both gain and loss conditions. The SME for a given condition is simply the standard deviation of these 1000 scores.

### 2.4 Code availability

**Fig. 2:**
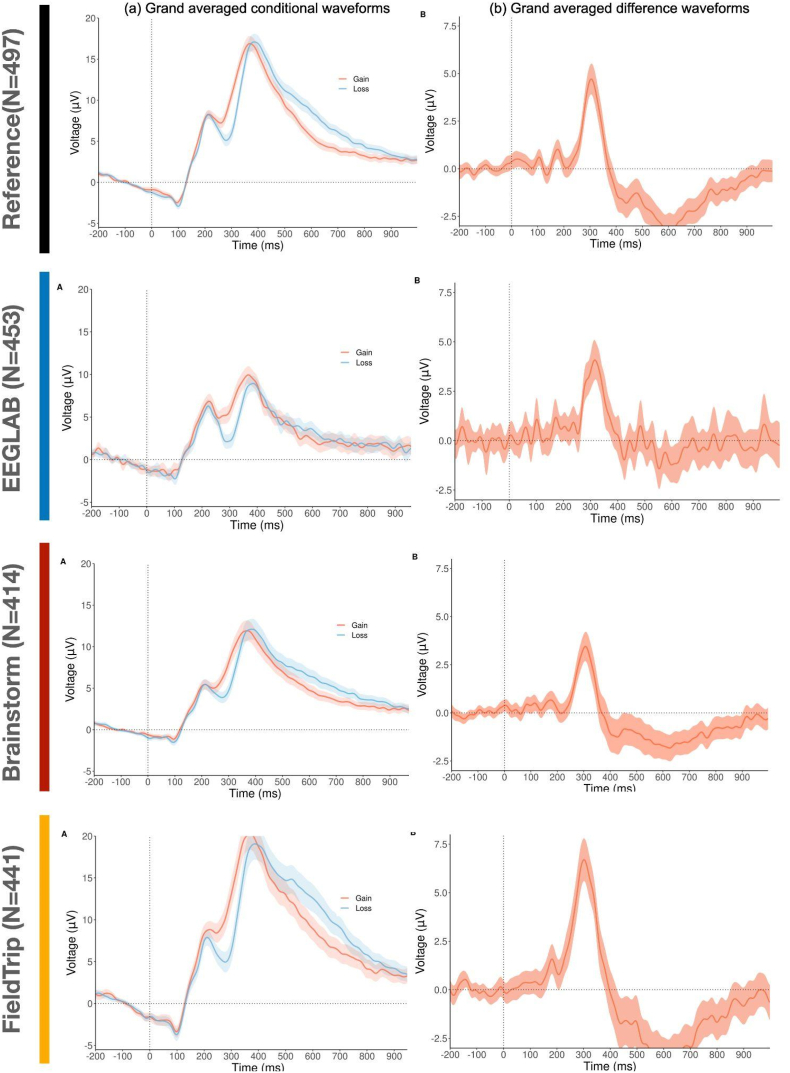
ERP waveforms at electrode FCz illustrating the reward positivity after preprocessing by: the reference code, EEGLAB, Brainstorm and FieldTrip. (a) Grand averaged conditional waveforms (ERP averaged across all subjects) with 95% confidence intervals, (b) grand averaged difference waveform with 95% confidence intervals. These subfigures are reproduced from Fig. 3 (parts a and b) illustrated in (Williams et al., 2021).

**Fig. 3:**
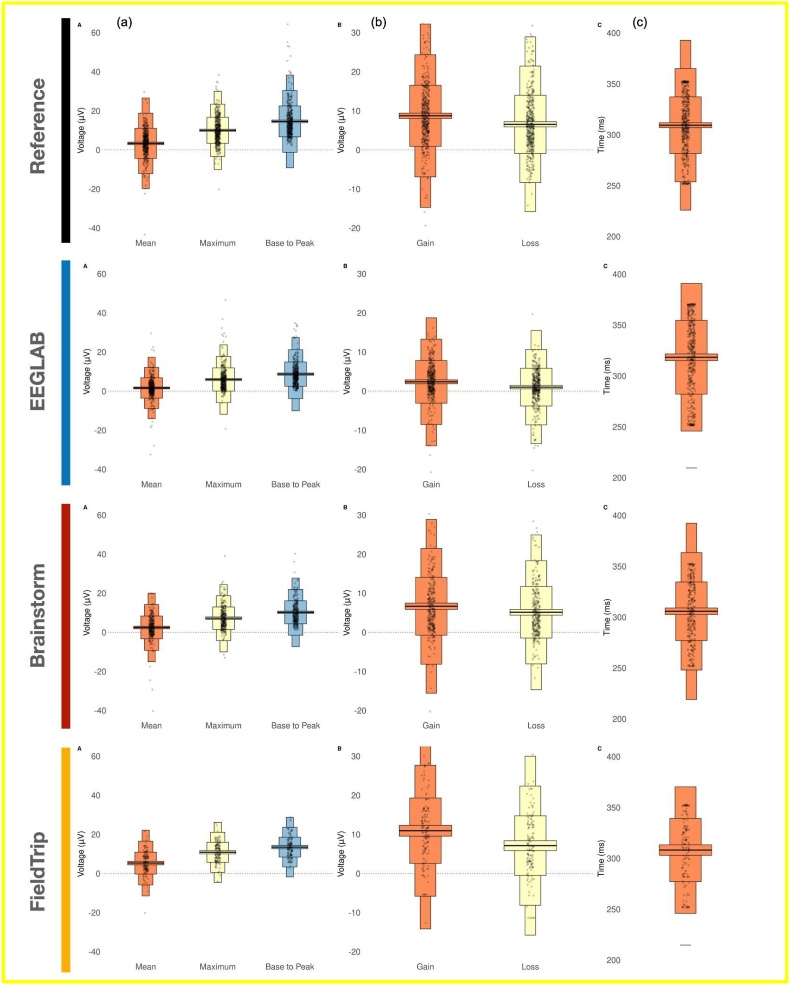
The metrics distribution across all participants for the different preprocessing software tools. (a) The features calculated on the difference ERP, (b) conditional amplitudes for the mean peak measure, and (c) peak latency of the reward positivity (difference ERP). Each black dot represents a participant's data and the middle black lines represent the mean across participants. These subfigures are a reproduction of Fig. 3 (part a,b and c) illustrated in (Williams et al., 2021).

**Fig. 4:**
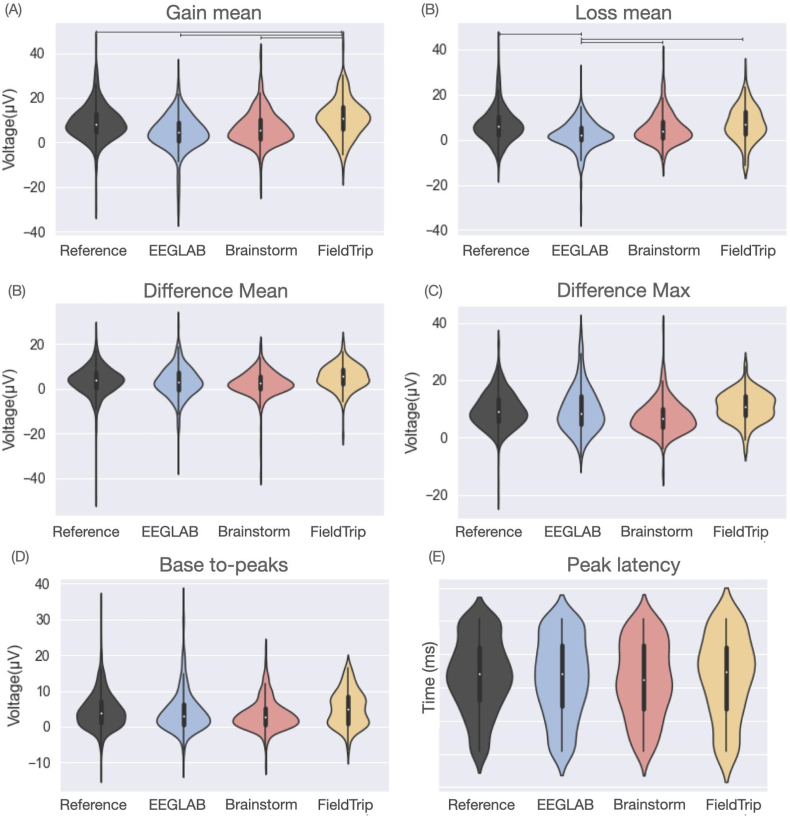
The violin plots showing the software distribution across subjects of the quantitative measures. A line between two violins denotes a statistical difference between their corresponding values.

**Fig. 5:**
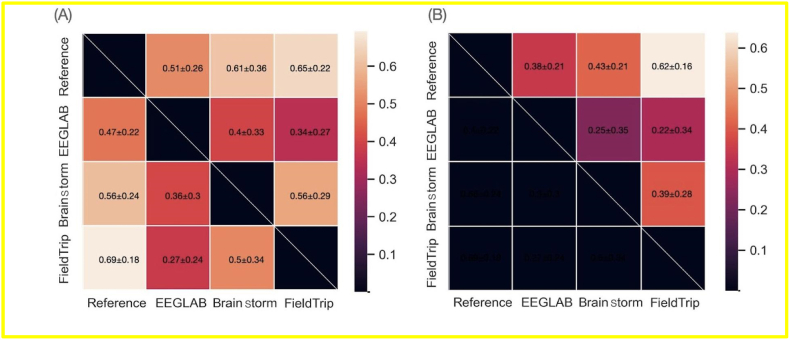
(A) The similarity matrix between the gain and loss ERPs obtained by the different software tools. The upper triangular part of the matrix corresponds to the gain condition while the lower part corresponds to the loss condition. (B) The similarity matrix between the difference ERP obtained by the different tools.

Codes supporting the results of this study are available at https://github.com/Inria-Empenn/EEG_preprocessing (released on Zenodo, doi: 10.5281/zenodo.6918329). All the preprocessing codes were written in Matlab (2018)). The visualizations of ERP waveforms (Fig. 2) and the quantitative features (Fig. 3) were done in R (R Core Team, 2020). Seaborn was used to illustrate the comparisons between the software distribution of the quantitative measures (Fig. 4), and the similarity matrix between software tools (Fig. 5). Other visualizations and statistical assessments were conducted using Matlab.

## 3 Results

### 3.1 Reproduction of the main findings

We observed a good degree of consistency between the ERP results published in the original paper and those reproduced using the script provided but excluding the ICA step (see Methods “Original preprocessing pipeline” for more details). Fig. S1 illustrates the difference between ERP waveforms at electrode FCz obtained by the reference script, with and without blink correction using ICA. In both cases, positive and negative deflections at the same peak latencies were observed. However, the peak amplitudes of the ERPs evoked by gain and loss conditions, observed at 400 ms latency were higher to those revealed when eye blinks were removed using ICA compared to the original paper's results. Table 1 and Table 2 report the descriptive statistics and the effect size of difference and conditional amplitudes of the ERPs. The same conclusions regarding the effect size related to the maximum and base to peak measures of the difference ERP, and the mean measure of the ERPs evoked by conditions were derived.

**Table 1:** Mean, maximum, base to peak and the effect size of the reward positivity (the difference ERP) for the reference paper as well as the three studied software packages EEGLAB Brainstorm and FieldTrip. The reported mean (in μV), standard deviation (in μV) and Cohen's d values were computed across subjects. This table is reproduced from Table 1 (first 3 rows) reported in (Williams et al., 2021).

		Mean [95% CI]	Standard deviation	Cohen's d [95% CI]
**Original paper (**Williams et al. 2021**)**	**Mean**	3.70 μV [3.34 μV, 4.07 μV]	4.11 μV	0.90 [0.77, 1.03]
	**Maximum**	7.82 μV [7.42 μV, 8.23 μV]	4.59 μV	1.71 [1.56, 1.85]
	**Base to peak**	10.52 μV [10.12 μV, 10.91 μV]	4.49 μV	2.34 [2.18, 2.50]
**Reference**	**Mean**	3.45 μV [ 2.75 μV, 4.15 μV]	7.94 μV	0.62 [0.48, 0.75 ]
	**Maximum**	9.73 μV [ 9.17 μV, 10.28 μV]	6.31 μV	2.18 [1.95, 2.41]
	**Base to peak**	14.00 μV [13.34 μV, 14.64 μV]	7.36 μV	2.69 [2.42, 2.96]
**EEGLAB**	**Mean**	2.40 μV [2.12 μV, 3.21 μV]	5.09 μV	0.68 [0.51, 0.77]
	**Maximum**	8.67 μV [7.60 μV, 9.13 μV]	7.95 μV	1.45 [1.33, 1.54]
	**Base to peak**	12.72 μV [11.6 μV, 12.93 μV]	9.97 μV	1.69 [1.54, 1.76]
**Brainstorm**	**Mean**	2.48 μV [2.15 μV, 2.80 μV]	5.85 μV	0.63 [0.58, 0.85]
	**Maximum**	7.21 μV [7.01 μV, 7.54 μV]	5.70 μV	1.77 [1.65, 1.94]
	**Base to peak**	10.20 μV [9.10 μV, 11.2 μV]	5.85 μV	2.46 [2.10, 2.73]
**FieldTrip**	**Mean**	5.34 μV [5.1 μV, 5.67 μV]	5.59 μV	1.34 [1.16, 1.54]
	**Maximum**	10.85 μV [9.91 μV, 11.20 μV]	5.12 μV	2.99 [2.34, 3.20]
	**Base to peak**	13.49 μV [12.75 μV,14.25 μV]	5.07 μV	3.76 [3.22, 3.95]

**Table 2:** Effect size of the gain and loss ERP, using the meak peak measure, for all software. The reported mean (in μV), standard deviation (in μV) and Cohen's d values were computed across subjects. This table is reproduced from Table 2 (first two rows) reported in (Williams et al., 2021).

		Mean [95% CI]	Standard deviation	Cohen's d [95% CI]
**Original paper (**Williams et al. 2021**)**	**Gain**	8.02 μV [7.54 μV, 8.49 μV]	5.38 μV	1.49 [1.35, 1.63]
	**Loss**	4.96 μV [4.53 μV, 5.38 μV]	4.87 μV	1.02 [0.89, 1.15]
**Reference**	**Gain**	9.05 μV [8.37 μV, 9.70 μV]	7.80 μV	1.64 [1.45, 1.83]
	**Loss**	6.80 μV [6.11 μV, 7.50 μV]	7.84 μV	1.23 [1.06, 1.40]
**EEGLAB**	**Gain**	4.70 μV [4.21 μV, 5.10 μV]	5.02 μV	1.28 [1.10, 1.40]
	**Loss**	2.41 μV [2.22 μV, 2.57 μV]	4.32 μV	0.79 [0.66, 0.90]
**Brainstorm**	**Gain**	6.66 μV [6.32 μV, 6.87 μV]	7.41 μV	1.27 [7.54, 8.49]
	**Loss**	5.12 μV [4.96 μV, 5.22 μV]	6.60 μV	1.09 [0.85, 1.28]
**FieldTrip**	**Gain**	10.90 μV [9.56 μV, 11.21 μV]	8.37 μV	1.84 [1.43, 1.92]
	**Loss**	7.12 μV [7.00 μV, 8.49 μV]	7.62 μV	1.32 [0.81, 1.65]

In Fig. 2, we illustrate the ERP waveforms at electrode FCz reflecting the reward positivity, obtained when running the preprocessing code of the reference paper, EEGLAB, Brainstorm and Fieldtrip. The grand averaged ERPs shown in Fig. 2 were obtained after averaging all the clean epochs (kept after the artifactual trials removal step) of all subjects. Changes in the amplitude of the two peaks were noticed for the ERP of gain and loss conditions generated by EEGLAB, Brainstorm and Fieldtrip, when compared to the reference results. An important variability in the number of remained trials/subjects obtained after the preprocessing was observed between the software tools: the number of subjects with clean data was N = 497 for the reference preprocessing, N = 453 for EEGLAB, N = 414 for Brainstorm code, and N = 441 for FieldTrip code. Despite those differences, there was a good level of concordance between the ERPs of gain and loss conditions obtained in terms of the two peaks latencies seen respectively at 212 ms and 370 ms for all the software tools. In addition, the same waveform profile showing positive and negative deflections at specific times was visualized. For instance, according to the gain waveform, the first peak amplitude obtained by the reference pipeline (8.3 ± 0.8 μV) was higher than that obtained by EEGLAB (5.9 ± 0.8 μV) and Brainstorm (5.1 ± 1.2 μV) and lower than that obtained by Fieldtrip (8.9 ± 1.4 μV). The amplitude of the second peak observed was higher in the reference pipeline (17.8 ± 0.8 μV) compared to EEGLAB (10.1 μV ± 0.5) and Brainstorm (12.4 ± 0.7 μV) but lower compared to Fieldtrip (21.1 ± 2.1 μV). The same findings can be observed for the loss waveforms. One can also notice that, for all software tools, the gain waveform elicited higher amplitude than the loss waveform, the loss between 0 ms and 450 ms, whereas the opposite occurs between 450 ms and 1000 ms.

According to the grand averaged difference, the reward positivity peaked at a latency of 310 ms for all the different software tools. The peak voltage is increased in Fieldtrip (6.6 μV ± 2.1) and decreased in EEGLAB (4.1 ± 1 μV), Brainstorm (3.4 ± 1.1 μV) compared to the reference (4.8 ± 0.9 μV).

Looking at the quantitative measures, results show good consistency between the reference and the software tools (Fig. 3, Table 1, Table 2). More specifically, a large effect size (d > 0.8) is obtained when looking at the maximum and base to peak measures of the difference ERP obtained by all the software tools (Table 1, last column). For the mean peak metric, results of all software present a medium effect size (0.8 > d > 0.5) except for FieldTrip that elicited a large effect size (d > 0.8). The effect size of the mean peak related to the gain and loss ERPs were considered as large in all the software tools (Table 2).

We also compared the results obtained by the four toolbox packages when reproducing a modified pipeline in which the bad channel detection method proposed by (Williams et al., 2021) was replaced by an automatic detection of flat channels (see Materials and Methods section for more details). Fig. S2 and Fig. S3 illustrate the results in terms of ERP waveforms and metrics distributions, respectively. Consistency in the peak latencies and voltage deflections is remarked. EEGLAB shows the lowest ERP amplitudes compared to the other tools. One important remark is the dramatic decrease in the number of subjects kept after the pre-processing (N = 264 for the reference, N = 191 for EEGLAB, N = 213 for Brainstorm, N = 397 for FieldTrip) compared to that kept when the original bad channel detection was used (N = 497 for the reference, N = 453 for EEGLAB, N = 414 for Brainstorm, and N = 441 for FieldTrip). A good degree of agreement is observed in terms of the effect size elicited by the different metrics (Table S1, Table S2), except for EEGLAB that shows a medium effect size for the mean peak of loss condition (Table S2). We also tested the variability of results between software packages after regulating the trial rejection threshold to 200 μV instead of the 100 μV min-max criterion while using the flat channels detection method. Readers can refer to the supplementary information for more details (Fig. S4, Fig. S5, Table S3, Table S4). The number of subjects kept after the preprocessing increased compared to that obtained when using the 100 μV min-max criterion. In addition, EEGLAB is remarkably showing a decrease in the voltage amplitudes of gain and loss ERPs compared to all other tools.

### 3.2 Comparison across software

A significant statistical difference was observed between FieldTrip and all the other tools in terms of mean peak amplitude of the gain ERP (Fig. 4). EEGLAB showed significant statistical differences with all the other tools in terms of mean peak voltage of the loss ERP. Regarding the features derived from the difference ERP (maximum peak, difference peak, base-to-peak and peak location), no statistical differences were found between software tools.

Fig. 5 illustrates the similarity matrix between the gain and loss ERPs generated by the different software tools when taking into account all the EEG channels. For each participant, the similarity between the preprocessed ERPs obtained from two different software tools was calculated using Pearson's correlation averaged across all channels (see materials and methods). Between the three Matlab toolboxes, FieldTrip reached the highest similarity with the reference pipeline for both conditions (0.65 ± 0.22 for gain; 0.69 ± 0.18 for loss; 0.62 ± 0.16 for the difference), followed by Brainstorm (0.61 ± 0.36 for gain; 0.56 ± 0.24 for loss; 0.43 ± 0.21 for the difference) then EEGLAB (0.51 ± 0.26 for gain; 0.47 ± 0.22 for loss; 0.38 ± 0.21 for the difference). Between the three tested tools, the highest similarity is observed between Brainstorm and FieldTrip (0.56 ± 0.29 for gain, and 0.5 ± 0.34 for loss; 0.39 ± 0.28 for the difference). All the reported correlations are significant with p-value lower than 0.01.

**Fig. 6:**
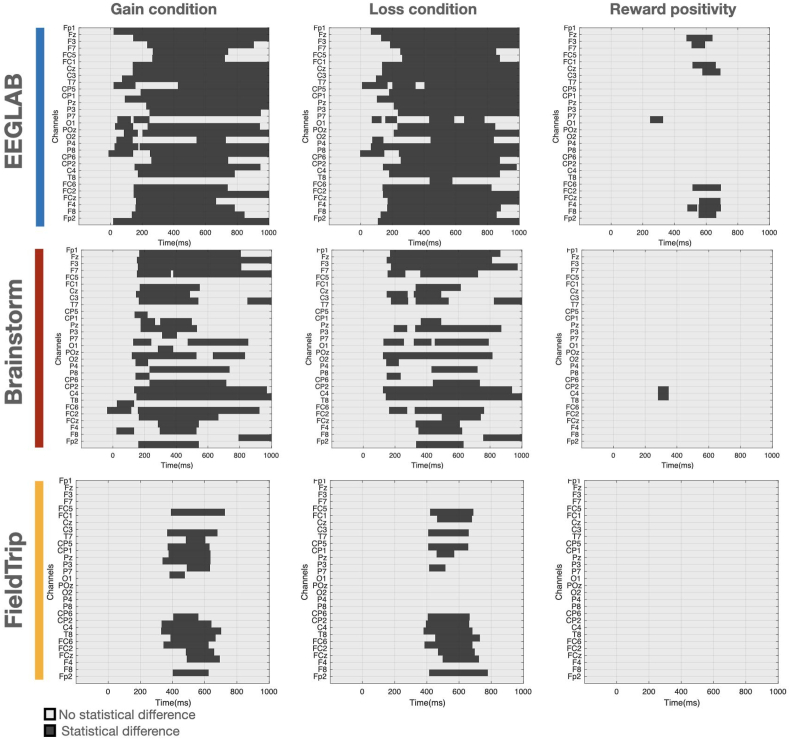
The statistical maps showing the differences between the results of each software tool and the reference at each millisecond and channel.

In addition, we investigated where and when the ERPs were statistically different by plotting the thresholded statistical map (time x channels). To do this, we quantified the statistical difference between the subjects' distribution of ERPs obtained from the reference pipeline and each of the tested tools, at each time sample and each channel using cluster-based permutation test (see materials and methods). In line with the previous findings, Fig. 6 highlights that EEGLAB shows the highest statistical differences compared to the reference results. One can also remark that EEGLAB statistical differences are distributed along the time axis starting from 200 ms to 1000 ms after the stimulus. The major statistical differences between FieldTrip and the reference results were revealed between 400 ms and 600 ms, at some EEG channels adequately. While both the gain and loss conditions showed important differences for all three software packages compared to the reference paper, there were only limited areas of significant differences in the reward positivity (i.e. ERP difference between gain and loss conditions). The statistical maps showing the cross-package differences (Fig. S15) show that FieldTrip and Brainstorm have the least number of clusters in time and channels. This is consistent with the similarity results measured in terms of correlations between software tools in Fig. 5.

The statistical analysis between metrics distributions did not show any significant difference between tools in either the gain and loss ERP metrics nor the difference ERP metrics when the bad channel detection method was replaced by a flat channel detection (Fig. S6). Compared to the original pipeline, fewer clusters of time/channels were revealed as significant when quantifying the statistical differences between ERP distributions (Fig. S7). The same observation was detected when exploring the cross-package differences (Fig. S16).

## 4 Discussion

A large range of techniques and tools are now available to process a single EEG dataset. This high analytical flexibility, reflected by the large number of choices made during the data preprocessing and analysis workflow, can be problematic as it can yield variability in research outcomes. Therefore, it is important to particularly understand the impact of the preprocessing methods, software package, software version and even the operating system on the reproducibility of the final research outcome of a study.

Here, we were interested in exploring the impact of the preprocessing software on the ERP derived from EEG data of 500 participants performing a simple gambling task as originally published by (Williams et al., 2021). The degree of agreement across software packages was good in terms of peak latencies and the general profile of ERP waveforms. In addition, the majority of the tested software tools obtained similar effect size estimates related to specific ERP features. Results show that differences between tools is lower in examining the contrast between gain and loss conditions than in examining absolute ERPs. However, remaining variability was also observed between software packages. This variability was reflected by the number of clean trials kept to compute the grand averaged ERPs (see Table S5), the peak voltages, the width of the confidence interval, and the statistical differences at particular channels and time instants (due to differences in absolute voltage values). Among the tested software tools used to reproduce the same preprocessing pipeline published by (Williams et al., 2021), EEGLAB seems to generate results with the lowest similarity when compared to the original ones while FieldTrip generates results with the highest similarity. However, it is noteworthy to clarify that we do not consider that the reference results obtained by the original script are better than those obtained by the other software tools in terms of the quality of the preprocessed signals. The objective of the current study is not to favor any software tool over another or to recommend the ‘best’ preprocessing tool, but rather, to illuminate and quantify differences that can be generated by different software tools on the same database The variations observed across tools can be related to several factors that are implicated in the preprocessing steps applied in each software. Results are discussed hereafter.

### 4.1 Influencing factors

In this study, our objective was to re-analyze the same data originally published and preprocessed in (Williams et al., 2021) using EEGLAB, Brainstorm and FieldTrip following the same original preprocessing workflow. Our intent was to fully automate all the preprocessing operations avoiding any manual intervention as much as practicable. Computationally, the workflow in each software was designed as a sequence of steps that are combined so that the intermediate outputs from one step directly feed as inputs into the next step. Notably, while all the tested software packages were purportedly replicating the same preprocessing steps, it was often impossible to exactly adapt the same methods and parameters used in the reference paper due to software implementation and configuration choices.

For instance, the band-pass filter cannot be configured in EEGLAB and Brainstorm to have the same type (Butterworth) and order used in the original paper. In addition, the gradient criteria adapted by the original study to detect the bad epochs is not supported by any of the tested software. EEG software packages such as EEGLAB and Brainstorm can choose to restrict the range of parameters that can be freely set by users in order to help practitioners by limiting the choice they have to make to perform their analysis.

Among the multiple influencing factors, the filter choice has a substantial impact on the resultant preprocessed signals. Conceptually, as no ideal filter exists, each filter (with the variation of type, order …) affects the temporal structure of EEG signals in both amplitude and phase (Rousselet, 2012; Vanrullen, 2011; Widmann and Schröger, 2012). Fig. S8 shows that the Butterworth filter used in reference and FieldTrip pipelines provide flat passband coming at a price of a broad transition band. The FIR filters implemented by EEGLAB and Brainstorm provide a narrow transition band coming at the cost of ripples in the stop-band. Regarding phase shifts, FIR filters generate equal delay at all frequencies and thus the signal shape will not be influenced by phase shifts. In contrast, different frequencies will appear at the filtered signal derived from the Butterworth filter with a different shift in phase. To better understand the effect of filters on the filtered signals, we illustrate an example of the filtered EEG signals obtained by the different tools for a random subject, and an example of their corresponding PSD (Fig. S9). It can be noticed from Fig. S9A that the filtered signal obtained using Brainstorm and EEGLAB shows higher peak-to-peak amplitudes compared to the filtered signals obtained using the reference and the FieldTrip filters. Fig. S9B reveals that while EEGLAB and Brainstorm directly drop the power of undesired frequencies (>30 Hz), the Butterworth filter used in the reference and FieldTrip scripts gradually attenuate the power of these amplitudes. The impact of the filters is directly reflected by the number of subjects and trials kept after trial rejection, as this latter is mainly based on the peak-to-peak criterion. This may explain the reason why Brainstorm has the lowest number of trials and subjects, followed by EEGLAB, FieldTrip and the reference tools (see Table S5). To better understand the effect of the filter type selected by (Williams et al., 2021) on the results, we filtered the raw signals of the 500 participants using the four filters: the Butterworth filter from the reference paper, EEGLAB FIR filter, Brainstorm FIR filter, and FieldTrip Butterworth filter. Then, for each participant, we assessed the correlation between the filtered signals derived between each pair of tools. Fig. S14A shows the matrix reporting the correlation values averaged across all participants. FieldTrip reached the highest correlation (r = 0.99 ± 0.0007) with the filtered signals generated by the reference filter, followed by EEGLAB (r = 0.88 ± 0.06) then Brainstorm (r = 0.71 ± 0.12). One important remark is that the correlation between EEGLAB and Brainstorm (that use the same type of filter - FIR), is lower than that obtained between the reference/FieldTrip and Brainstorm. This means that the effect of the filter on the signal amplitude also exists even when using similar types of filters showing comparable responses (Fig. S8). However, the observed impact on continuous EEG signals does not necessarily imply a similar impact on ERP-derived signals. Therefore, we also explored whether the use of the reference filter has led to the major differences in the ERP amplitudes or not. We thus compared the results obtained by the reference script with the Butterworth filter to those obtained using the same script with only the filter replaced by the FIR filter (as designed by EEGLAB). In Fig. S10, we show the results obtained after preprocessing following the same channel detection method reported in (Williams et al., 2021). By using a simple flat channel detection method, we obtained the results illustrated in Fig. S11. Fig. S10 and Fig. S11 show no remarkable visual difference between the ERP waveforms. Consistently, no significant difference was reported in the quantitative measures, neither in the amplitudes of the ERP waveforms at any channel or time sample when the permutation test was performed. It is important to mention here that we do not aim to evaluate the performance and suitability of the applied filters, but to examine how each filter has impacted the resultant ERP signals. Additionally, the way trials were rejected may also lead to discrepancy in the results. Compared to other tools, EEGLAB does not reject trials based on a peak-to-peak voltage threshold but rather on minimum and maximum thresholds. This criterion might reject trials that are exceeding the maximum or minimum thresholds but not necessarily exceeding the peak-to-peak threshold.

More crucially, the method proposed by the original paper to detect the bad channels contributed to the major disparities between tools. In fact, this method implies applying a set of processing methods (including re-referencing, filtering, epoching, bad trial detection) in order to detect the bad channels as those showing a trial rejection rate exceeding 40%. The same set of processing methods are repeated in a second data pass (see materials and methods section for more details). This means that the variability induced by the repeated processing substeps (mainly the filtering and the trial rejection methods discussed above) affects results twice: the first impact occurs when detecting the bad channels, and the second one occurs when the final ERP waveforms are constructed. To be more precise, the number of bad channels detected per subject following the original pipeline greatly differs between tools. For the reference script, the number of rejected channels is 7 ± 5 channels depending on the subject, 10 ± 7 channels by EEGLAB, 13 ± 5 channels by Brainstorm and 6 ± 7 channels for FieldTrip**.** Obviously, the interpolation of a different number of electrodes is an additional influencing factor impacting the ultimate results.

As it is uncommon to process data twice as done in the original pipeline, we tested the variability between software tools when the first processing pass was replaced by a traditional channel detection method (i.e. see “Modified preprocessing pipelines” in the Methods section). In this case, only one pass was included in the preprocessing pipeline. Our findings show that variability between software tools decreased. This is because the flat channel detection methods in all the different packages have led to the identification of identical bad channels. The number of rejected bad channels is 2 ± 1 channels for all tools. In contrast, the method proposed by the original paper to detect the bad channels has led to differences in the detected bad channels (as previously noted). Since the pre-processing outcome will greatly differ depending on the bad channels interpolated, the consistency between results will increase when consistency in the detected bad channels is observed. This outlines that avoiding the repetition of signal processing functions, and using validated standardized pipelines recommended by the major software environments (rather than developing custom pipelines) can be an important approach to reduce analytical variability.

Moreover, the number of trials and subjects kept by all the software packages after the original preprocessing pipeline was much higher than that obtained using the traditional channel detection (flat channel detection method here). This is because the bad channels replaced by interpolated data by the original pipeline were determined in a way to have a low trial rejection rate (see materials and methods section for more details). To increase the number of ‘good’ trials used ultimately to reconstruct the ERPs, we regulated the trial rejection threshold used in the bad trial identification step. Instead of the 100 μV min-max criterion, we tested the variability of results between software tools when using the 200 μV min-max criterion. The corresponding results show an increase in the number of subjects and trials kept after preprocessing (see Table S5).

This highlights the need in future work for a multi-stage assessment of software differences, to examine which steps made the major difference in study's outcomes, and which steps were of less concern. In this study, we explored the independent effects of three of the factors that may affect the reproducibility of the preprocessed ERP: i-the filtering method, ii-the trial rejection method and, iii-the channel detection method. To explore this effect, the same data were set as inputs for the different methods, and the results were statistically compared using correlation measures (see supplementary information for more details about this analysis). When interpreting the results illustrated in Fig. S14, one could expect that the difference in the ERP obtained between EEGLAB and the reference is mostly due to the difference in the rejected number of trials, and the bad channels detected. In addition, the difference between Brainstorm and the reference is mostly due to the filter effect. As FieldTrip showed the highest correlations with the reference results when exploring the effects of the filtering, trial rejection and bad channel rejection methods, the derived ERPs were the most comparable to those obtained by the reference script as demonstrated in Fig. 5, Fig. 6.

### 4.2 Reproducibility in the neuroimaging field

The question of reproducibility and replicability is considerably gaining attention in the scientific community (Fidler and Wilcox, 2018; Munafò et al., 2020; Nosek et al., 2015). In the neuroimaging field, a recent study addressed the issue of analytical flexibility in fMRI research and its effects on the associated conclusions (Botvinik-Nezer et al., 2020). Using the same data, variability in results was reported in testing nine hypotheses across seventy independent teams. Inspired by this study, two recent initiatives have been made to test the effect of diversity of analysis pipelines and teams on EEG results. The ‘EEGManyPipelines’ (Algermissen et al., 2021) project and EEGManyLabs (Pavlov et al., 2021) were recently launched to involve many independent teams in analyzing the same data and testing a set of predefined hypotheses. Multiple EEG studies have also demonstrated that a study's outcomes are contingent on subjective decisions and factors selected in the EEG analysis, such as the EEG electrode density (Allouch et al., 2022; Lantz et al., 2003; Sohrabpour et al., 2015; Song et al., 2015), the preprocessing methods and parameters (Barban et al., 2021; Clayson et al., 2021; Robbins et al., 2020; Šoškić et al., 2022), the number of trials (Boudewyn et al., 2018), the filtering methods (Rousselet, 2012; Widmann and Schröger, 2012) and the specific parameters related to the EEG connectivity analysis (Allouch et al., 2022; Hassan et al., 2014). The variability in the software used in EEG analysis was tackled in a recent review that addresses the question of reproducibility and consistency of ERP studies, mainly focusing on the N400 component (Šoškić et al., 2021). In a sample of 132 ERP papers (Šoškić et al., 2021), reveals that the number of software tools used to perform the EEG analysis stages (from the presentation of stimulus to the statistical assessment) ranged from 8 to 17 options, and that such methodological decisions can induce substantial variability in the reported results, ultimately hindering research replicability. Using fMRI, many studies have quantified the impacts of the analysis software (Bowring et al., 2019; Li et al., 2021), the software version (Gronenschild et al., 2012) and the operating system (Glatard et al., 2015; Gronenschild et al., 2012) on results conducted on a single dataset. In the current study, we focused on examining whether it is possible to reproduce the same ERP results after preprocessing data with different software tools. To the best of our knowledge, the effect of the preprocessing software on the same EEG dataset has never been studied before. This current study has not only provided a validation of EEGLAB, Brainstorm and FieldTrip but also it contributed to better understand the possible discrepancies in results generated by different EEG studies.

### 4.3 Methodological considerations

In this work, we attempted to reproduce using different software tools the same preprocessing pipeline initially proposed by (Williams et al., 2021). This was carefully done by conserving, as much as possible, the same steps along with their related parameters (band-pass filter cut-off frequencies, baseline duration, reference electrodes.etc) and order. Nevertheless, the derived signals and results might be also sensitive to other factors that were not investigated in this study. For instance, the parameters used to detect the artifactual channels were set to the default or to the most commonly used values as recommended by each toolbox (such as the window length in which signals are completely flat, the correlation with neighbors threshold and other criteria). An interesting future prospect would be testing the consistency of results when varying these factors.

To validate the hypotheses supported by the reference paper exploring the same dataset, we compared the results generated by each software to those originally published by (Williams et al., 2021). The cross-software discrepancies were also quantified. Another issue that may be of great interest to be investigated is to evaluate the feasibility of each preprocessing tool in generating reliable signals with good data quality. This could be done by comparing results to ground-truth data generated ideally by a computational model of electrophysiological signals such as neural-mass models (Bensaid et al., 2019) or multivariate autoregressive models (Haufe and Ewald, 2019).

In order to conduct the comparative analysis between the results generated by the different software tools, we used several quantification metrics to measure the consistency/discrepancy of ERP waveforms and their related characteristics. We mainly relied on ERP as the main objective of this work was to reproduce and validate the results published by (Williams et al., 2021) studying the reward positivity. However, it is commonly known that ERP strategy is based on an across-trial averaging which increases the signal-to-noise ratio, and discards much information in single-trial EEG activities. Thus, we are aware that the consistency of results may greatly differ if the analysis was performed on the continuous preprocessed EEG instead of ERPs computed after averaging a large number of epochs. In addition, considering smaller sample sizes could also lead to higher levels of cross-software variability as previous literature has outlined how variability induced by different pipelines decreases with higher signal-to-noise ratio (e.g. see (Li et al., 2021) for an example with resting rate fMRI of various acquisition durations).

A crucial step in a preprocessing pipeline is the artifact removal of various contaminations. Numerous techniques have been proposed, ranging from regression, Blind source separation including Independent and Principal Component Analyses, to Empirical-mode Decomposition and others (readers can refer to (Jiang et al., 2019) for a review). In the original pipeline, ICA-based eye blinks removal was applied by visually detecting the artifactual components. This step (which required manual intervention) was eluded in our study in order to prevent the impact of inter-rater variability on results. But the results may also be affected by the selected cleaning method as highlighted in (Barban et al., 2021; Clayson et al., 2021; Robbins et al., 2020) where the variability across different Blind source separation techniques was explored.

One important question that may arise when examining the difference in the accepted number of trials used to average ERP, is to what extent this variation affects the data quality. Thus, we compared the Standardized Measurement Error (SME) metric between the ERP obtained by the different tools. As reported in (Luck et al., 2021), the SME is the standard error of measurement for a particular score. Here, the score we chose was the mean peak score (average of the voltages ±46 ms surrounding the peak location) as this score is commonly used in the context of reward positivity (Sambrook and Goslin, 2015). Fig. S13 shows the distributions between the SME distribution across subjects of the different software packages. Results show that the mean peak SME was the best (the lowest) for FieldTrip, followed by the reference, then EEGLAB then Brainstorm**.** A one-way ANOVA revealed that there was not a statistically significant difference in SME between the four tested tool with F(3,497) = 1.07, and p-value = 0.37 (greater than 0.05). These findings demonstrate that even with the difference in the number of accepted trials used in averaging ERPs, the data quality (measured by mean peak SME) was statistically similar. A possible interpretation of this result is that both gain and loss waveforms have a clear peak, on which noise in the data had low impact on the surrounding mean voltage score.

Among the available preprocessing tools used in EEG studies, we selected three of the most commonly used open-source software tools. In each software, we tried to follow, as much as possible, the same preprocessing workflow of (Williams et al., 2021) using the provided software functions. This led us to exclude other interesting packages that conduct a fully automatic preprocessing such as automagic (Pedroni et al., 2019), the Harvard Automated Preprocessing Pipeline for EEG (HAPPE) (Gabard-Durnam et al., 2018) and the Batch Electroencephalography Automated Processing Platform (BEAPP) (Levin et al., 2018) toolboxes. In other words, our inability to control or modify the inclusion and the order of the various preprocessing steps impedes these toolboxes to respect the same preprocessing pipeline we were trying to reproduce. Besides Matlab, it would be interesting to investigate and systematically quantify the differences of results generated by the MNE-python package (Gramfort et al., 2014). Despite the wide acceptance of MNE-Python in the scientific community, we limited our study to examine the variability of results obtained by the most commonly used Matlab-based tools. Thus, the three tested tools are developed under the same environment and using the same language. We would also highlight that the scope of the paper is limited to open-source packages although the ability of many distributed commercial software to reproduce the same preprocessing pipeline (such as BESA, Curry, and PRANA, Netstation, BrainVision Analyzer). Our choice relied on the opportunity provided by open-source tools to implement, customize and modify the script functions with no upfront financial costs.

In addition, it is unclear how findings reported in this paper would generalize to other datasets or experimental paradigms. Therefore, it would be interesting to evaluate the fluctuations of results on other datasets and tasks covering further preprocessing pipelines and steps. For instance, one may examine whether the effect of the preprocessing is more or less important in a task-free compared to task-related paradigms. In addition, one important preprocessing step that needs to be included in further preprocessing pipelines is the artifact detection method. Finally, a multiverse analysis (https://journals.sagepub.com/doi/10.1177/1745691616658637) of a data preprocessing pipeline examining the impact of a large set of analytic choices might be also useful to researchers to determine the effects of different decisions.

## 5 Conclusions

This study sheds light on how the software tool used to preprocess EEG signals impacts the analysis results and conclusions. EEGLAB, Brainstorm and FieldTrip were used to reproduce the same preprocessing pipeline as a published EEG study performed on 500 participants. While the three software tools succeeded to infer the same conclusion of the original publication regarding the effect size estimates related to the derived ERP features and the peak latency of the obtained ERP, we observed significant differences in terms of the observed absolute voltage between EEGLAB, Brainstorm and Fieldtrip results, as well as between each of the software tools and the original results. Minor statistical differences were detected between tools in terms of ERP difference between conditions. In addition, the use of standardized pipelines documented in major software environments is more recommended to reduce variability in results, rather than developing custom pipelines. To better understand the variability induced by the software tool, further comparative studies should be conducted to examine the effects on the continuous EEG signals instead of ERP signals. In addition, more in-depth analysis is recommended in order to identify the critical steps and factors that lead the most to the variability observed.

## Declaration of competing interest

The authors declare that they have no known competing financial interests or personal relationships that could have appeared to influence the work reported in this paper.

## Data Availability

I have shared the links to data and code used
