# Supplementary material for: Successful reproduction of a large EEG study across software packages
Source: Neuroimage Rep. 2023 May 27;3(2):100169. doi: 10.1016/j.ynirp.2023.100169 (PMC12172740; doi:10.1016/j.ynirp.2023.100169)
Supplement: Multimedia component 1 [file mmc1.pdf]

Supplementary materials for

Successful reproduction of a large EEG study across software packages

Aya Kabbara[1,2], Nina Forde[3], Camille Maumet[3*], Mahmoud Hassan[2,4*]

[1] LASeR - Lebanese Association for Scientific Research, Tripoli, Lebanon

[2] MINDig, F-35000 Rennes, France

[3] Inria, Univ Rennes, CNRS, Inserm, IRISA UMR 6074, Empenn ERL U 1228, Rennes, France

[4] School of Science and Engineering, Reykjavik University, Reykjavik, Iceland.

[*] Equally contributed

Corresponding author: camille.maumet@inria.fr

After removing correction using ICA **Before removing correction using ICA**

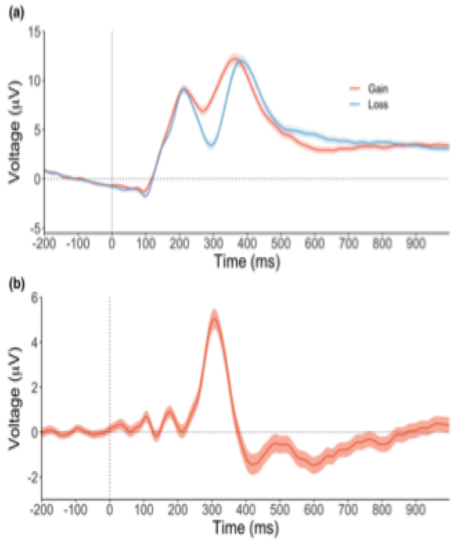

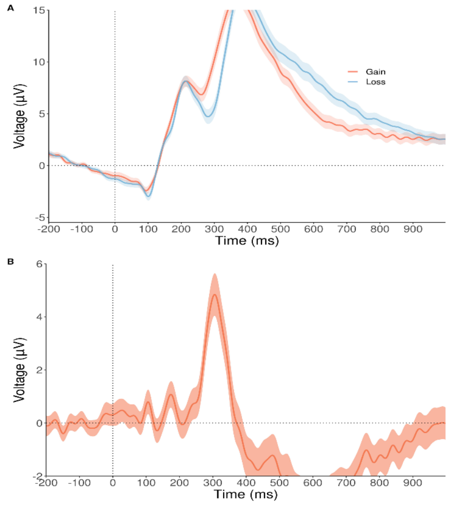

Figure S1. The difference between the ERP waveforms obtained using the reference script with and without the blink correction. A) conditional ERPs for gain and loss condition, B) difference ERP between gain and loss.

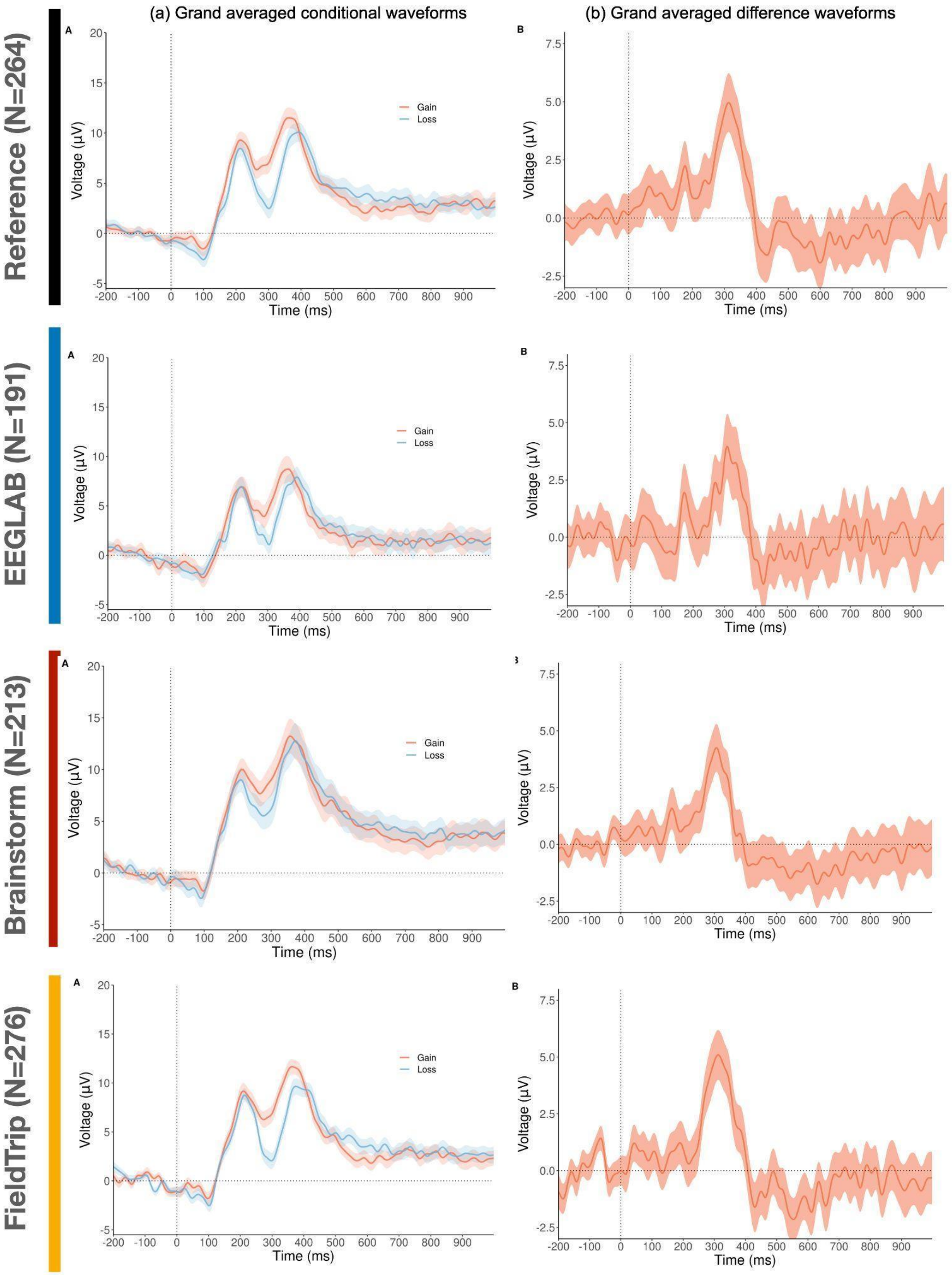
(a) Grand averaged conditional waveforms
(b) Grand averaged difference waveforms
Reference (N=264)
EEGLAB (N=191)
Brainstorm (N=213)
FieldTrip (N=276)
A
B
Gain
Loss
Voltage (µV)
Time (ms)
20
15
10
5
0
-5
7.5
5.0
2.5
0.0
-2.5
-200
-100
0
100
200
300
400
500
600
700
800
900

Figure S2. ERP waveforms at electrode FCz illustrating the reward positivity obtained by: the reference code, EEGLAB, Brainstorm and FieldTrip using the automatic way integrated in each tool to detect the flat channels with a trial rejection peak-to-peak threshold of 100 microVolts. (a) grand averaged conditional waveforms (ERP averaged across all subjects) with 95% confidence intervals, (b) grand averaged difference waveform with 95% confidence intervals. These subfigures are reproduced from Figure 3 illustrated in (Williams et al. 2021).

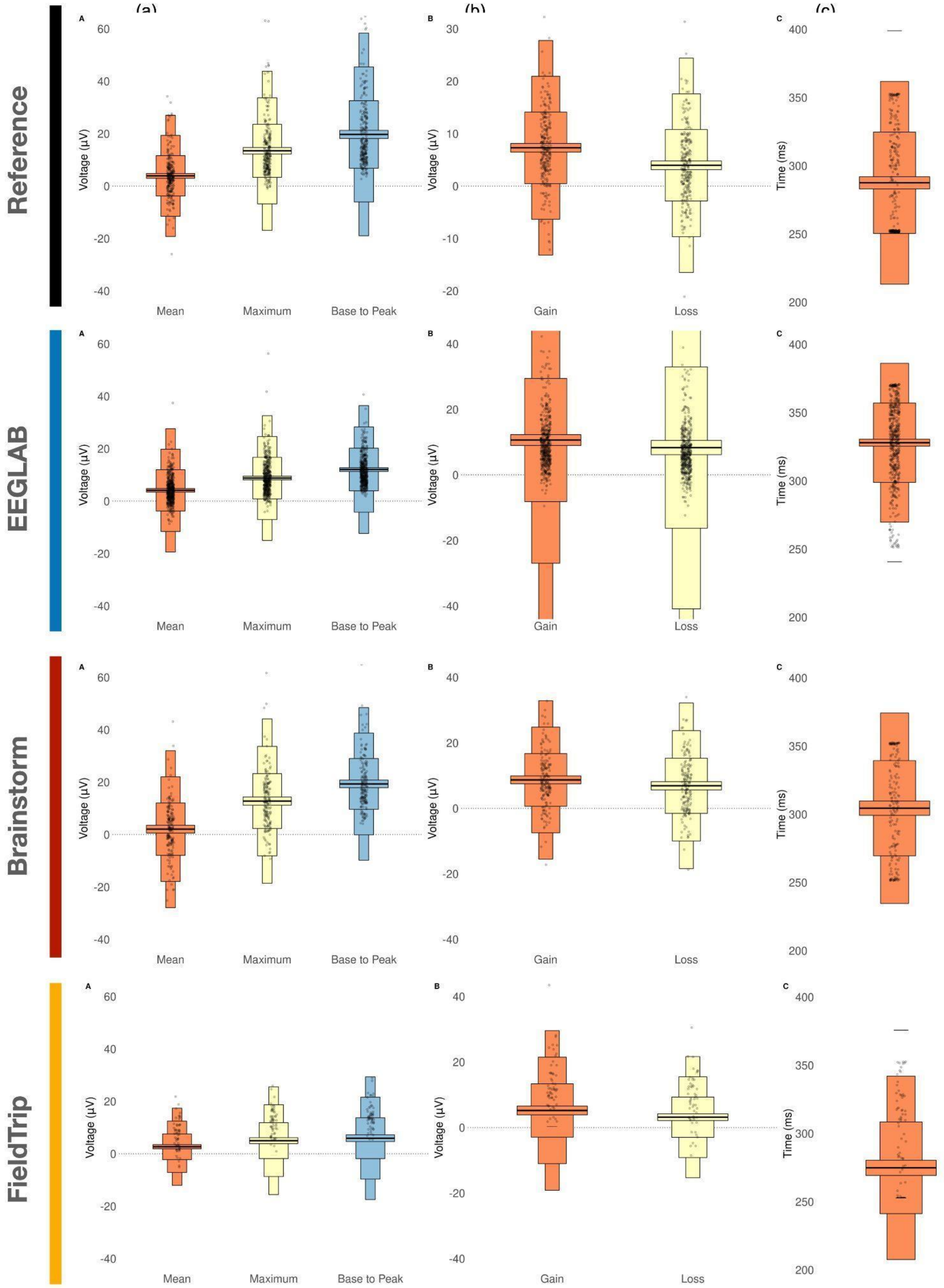

(a)
(b)
(c)
Reference
EEGLAB
Brainstorm
FieldTrip
A
B
C
Voltage (µV)
Time (ms)
Mean
Maximum
Base to Peak
Gain
Loss

Figure S3. The metrics distribution across all participants for the different preprocessing software tools using the automatic way integrated in each tool to detect the flat channels with a trial rejection peak-to-peak threshold of 100 microVolts. (a) The features calculated on the difference ERP, (b) conditional amplitudes for the mean peak measure, and (c) peak latency of the reward positivity (difference ERP). Each black dot represents a participant's data and the middle black lines represent the mean across participants. These subfigures are a reproduction of Figure 3 illustrated in (Williams et al. 2021).

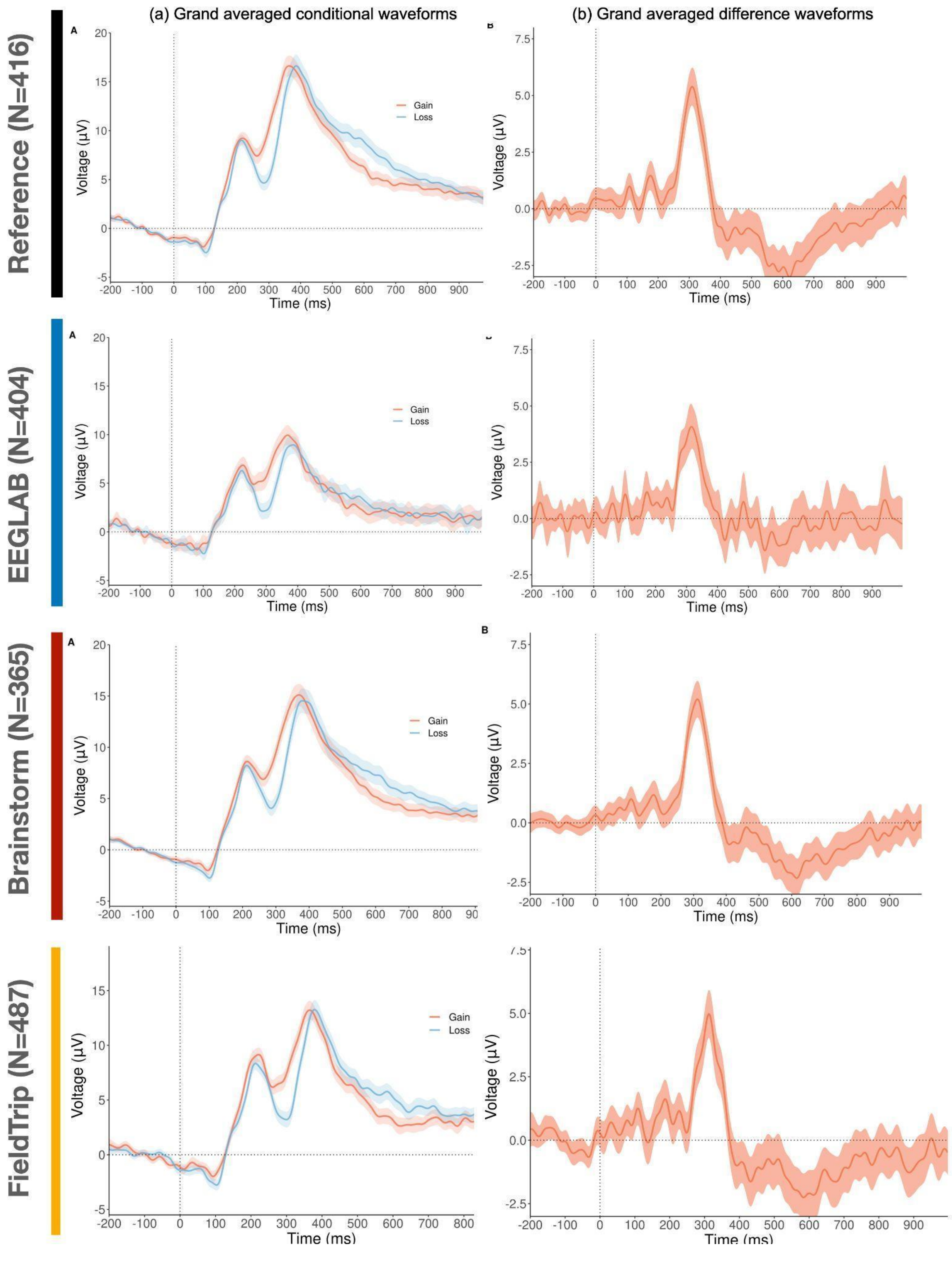

Figure S4. ERP waveforms at electrode FCz illustrating the reward positivity obtained by: the reference code, EEGLAB, Brainstorm and FieldTrip using the automatic way integrated in each

tool to detect the flat channels with a trial rejection peak-to-peak threshold of 200 microVolts. (a) grand averaged conditional waveforms (ERP averaged across all subjects) with 95% confidence intervals, (b) grand averaged difference waveform with 95% confidence intervals. These subfigures are reproduced from Figure 3 illustrated in (Williams et al. 2021).

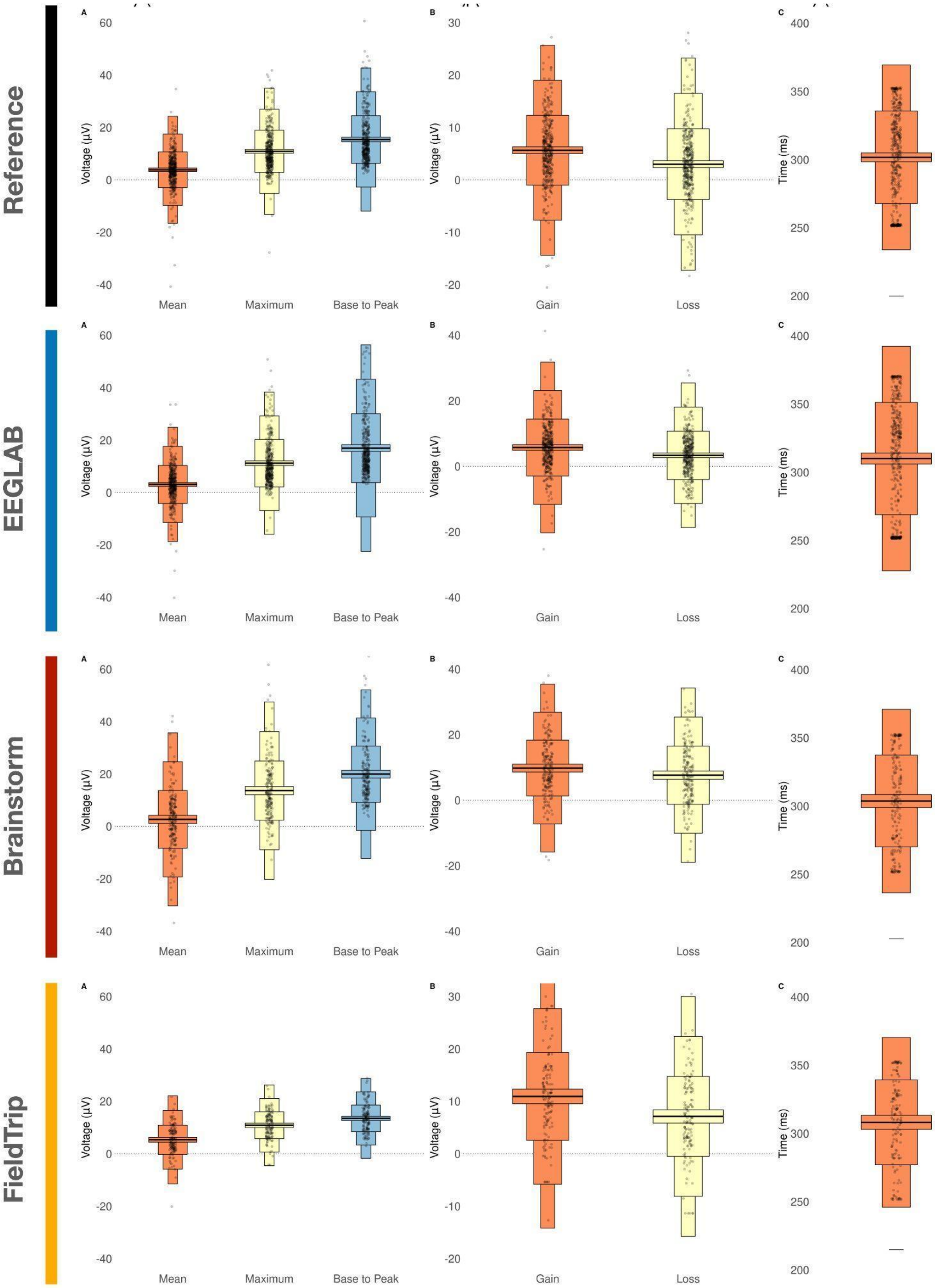
Reference
EEGLAB
Brainstorm
FieldTrip
A
B
C
Voltage (µV)
Time (ms)
Mean
Maximum
Base to Peak
Gain
Loss

Figure S5. The metrics distribution across all participants for the different preprocessing software tools using the automatic way integrated in each tool to detect the flat channels with a trial rejection peak-to-peak threshold of 200 microVolts. (a) The features calculated on the difference ERP, (b) conditional amplitudes for the mean peak measure, and (c) peak latency of the reward positivity (difference ERP). Each black dot represents a participant's data and the middle black lines represent the mean across participants. These subfigures are a reproduction of Figure 3 illustrated in (Williams et al. 2021).

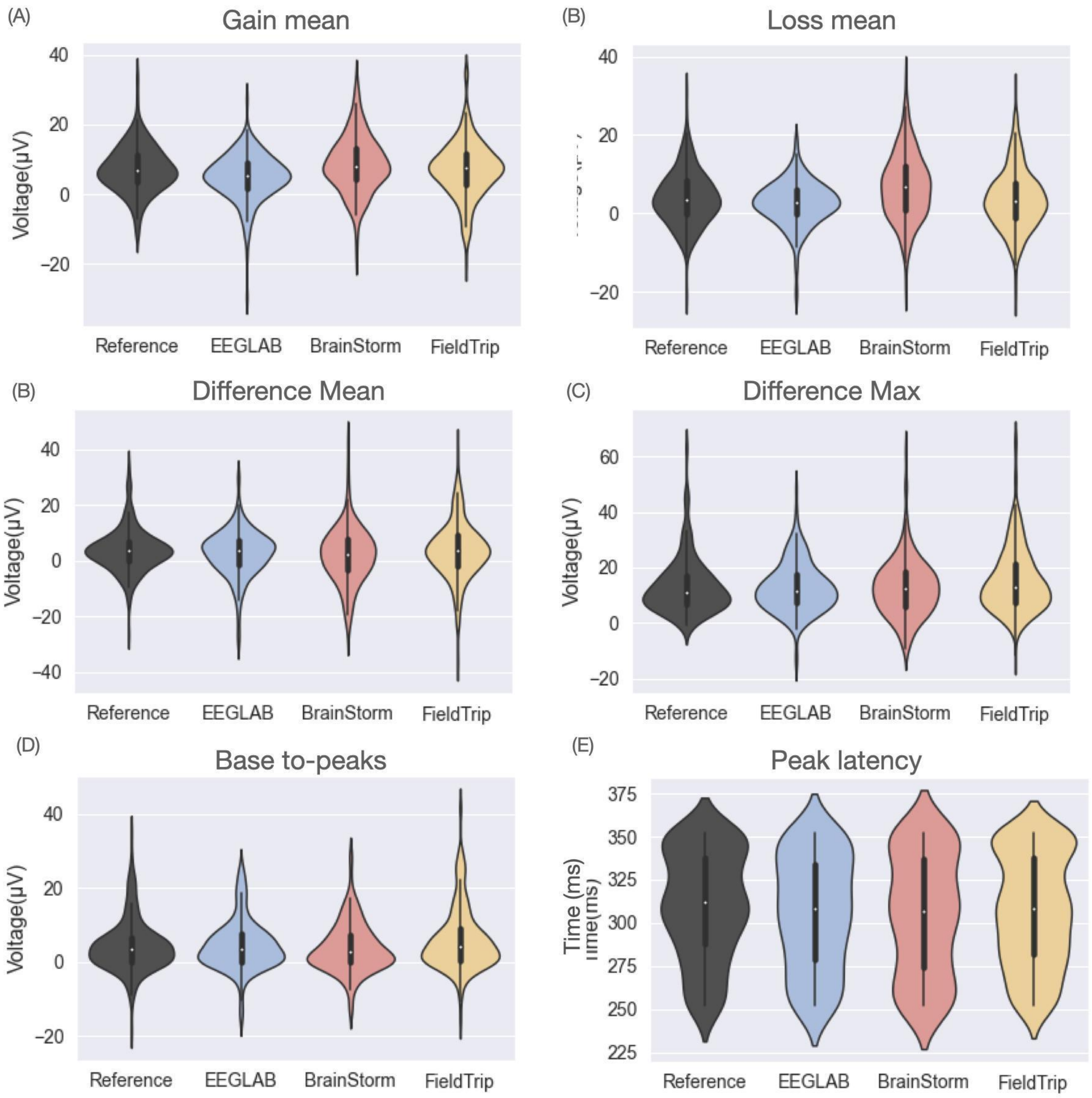

Figure S6. The violin plots showing the software distribution across subjects of the quantitative measures using the automatic way integrated in each tool to detect the flat channels with a trial rejection peak-to-peak threshold of 100 microVolts. A line between two violins denotes a statistical difference between their corresponding values.

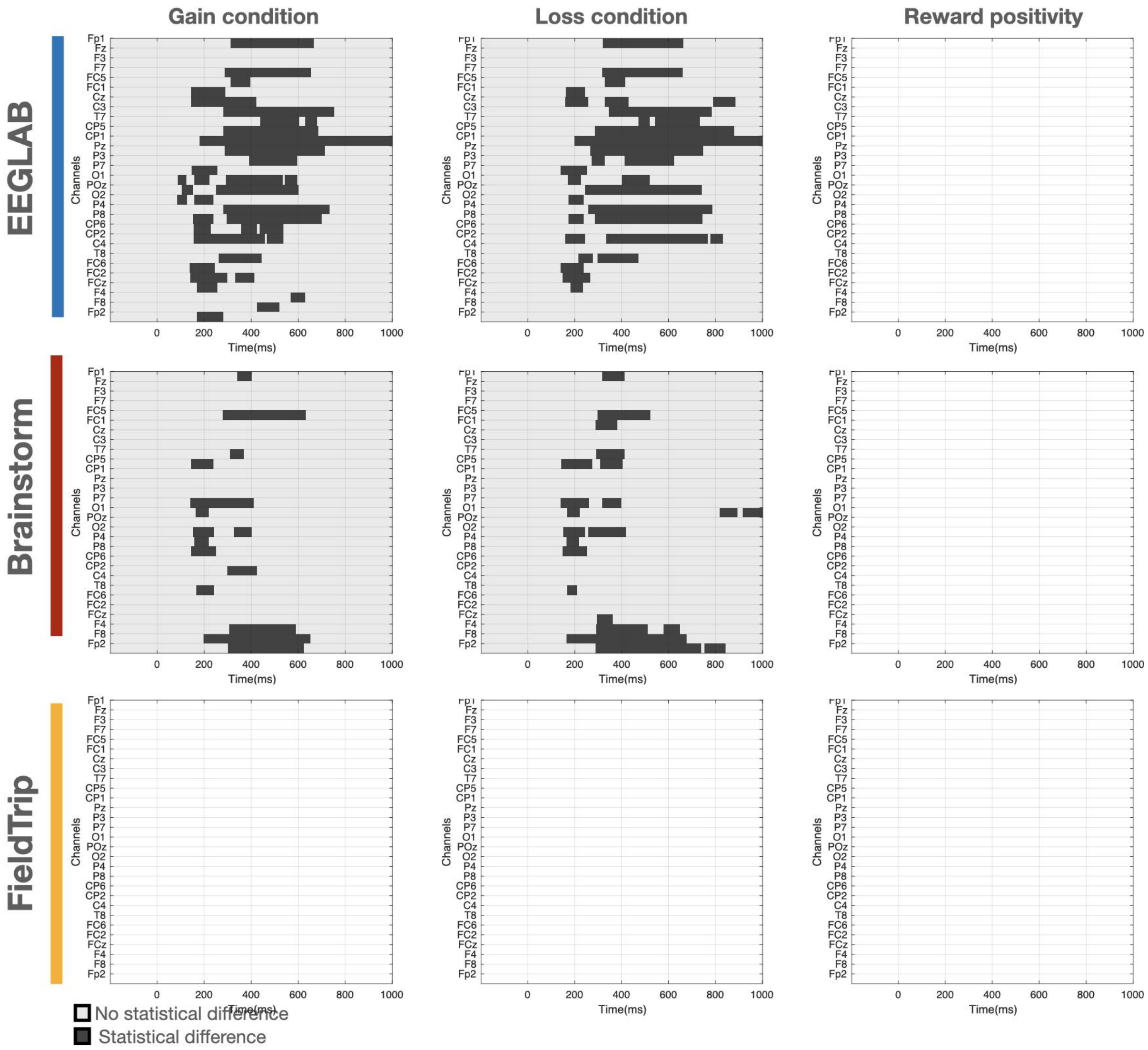

Figure S7. The statistical maps showing the differences between the results of each software tool and the reference at each timepoint and channel using the automatic way integrated in each tool to detect the flat channels with a trial rejection peak-to-peak threshold of 100 microVolts.

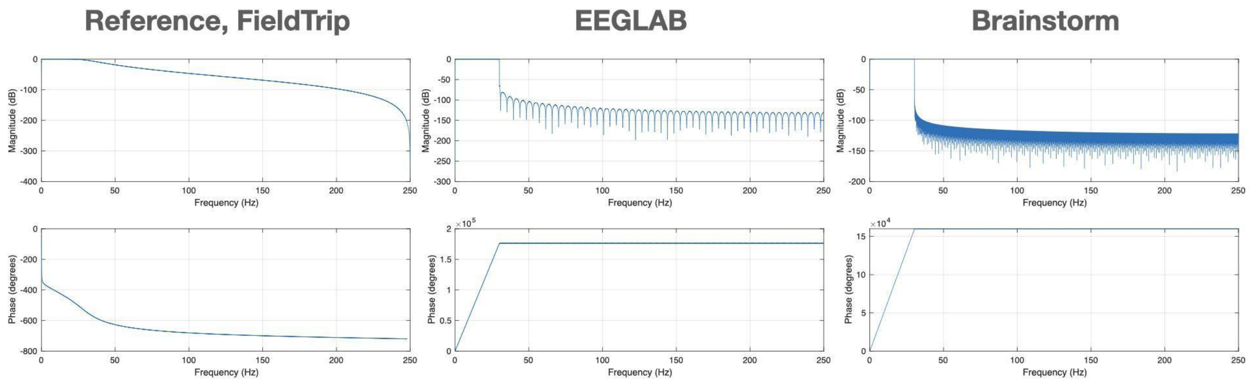

Figure S8. Frequency responses of the filters used in the reference and FieldTrip pipelines (IIR Butterworth), EEGLAB (FIR) and Brainstorm (FIR).

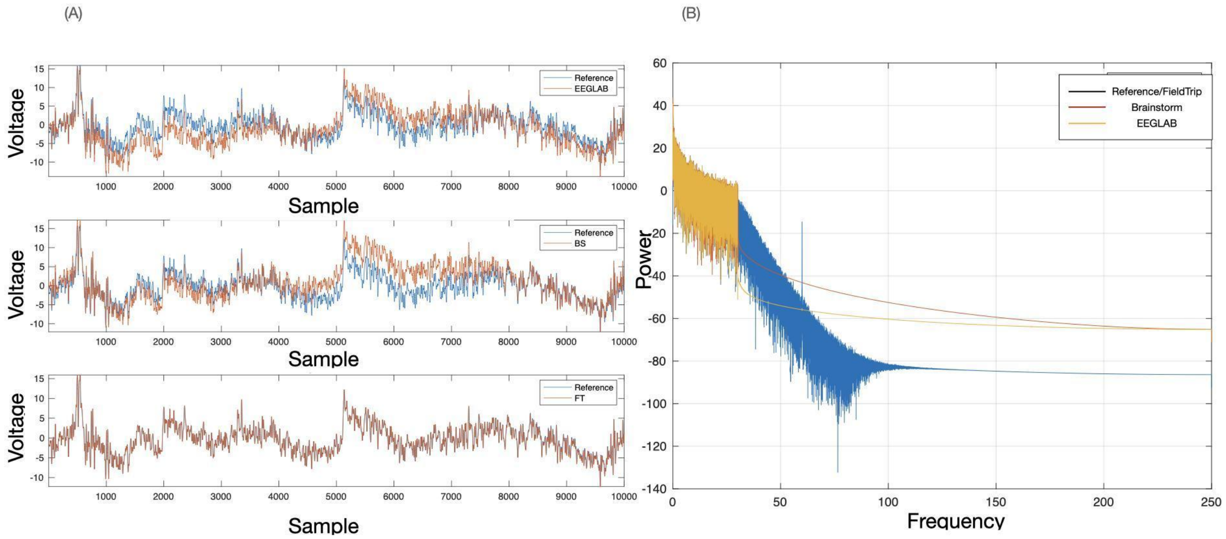

Figure S9. A) An example of filtered signals for a randomly selected subject, B) The corresponding power spectral density.

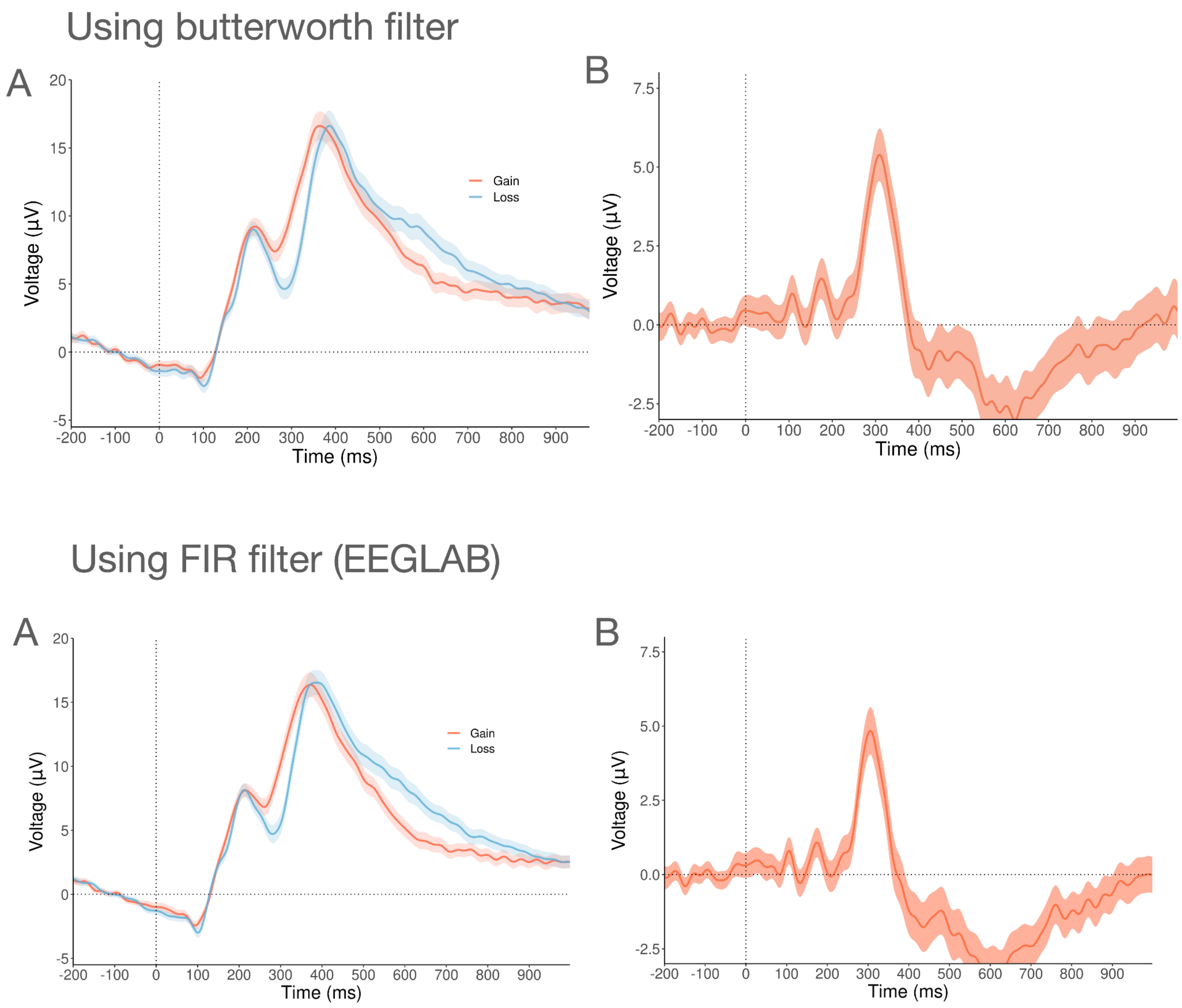

Figure S10. The difference between ERP waveforms after preprocessing by the reference code with the Butterworth filter (as in Williams et al. 2021) and with a FIR filter as designed in EEGLAB, using the automatic channel detection method. (A) grand averaged conditional waveforms (ERP averaged across all subjects) with 95% confidence intervals, (B) grand averaged difference waveform with 95% confidence intervals.

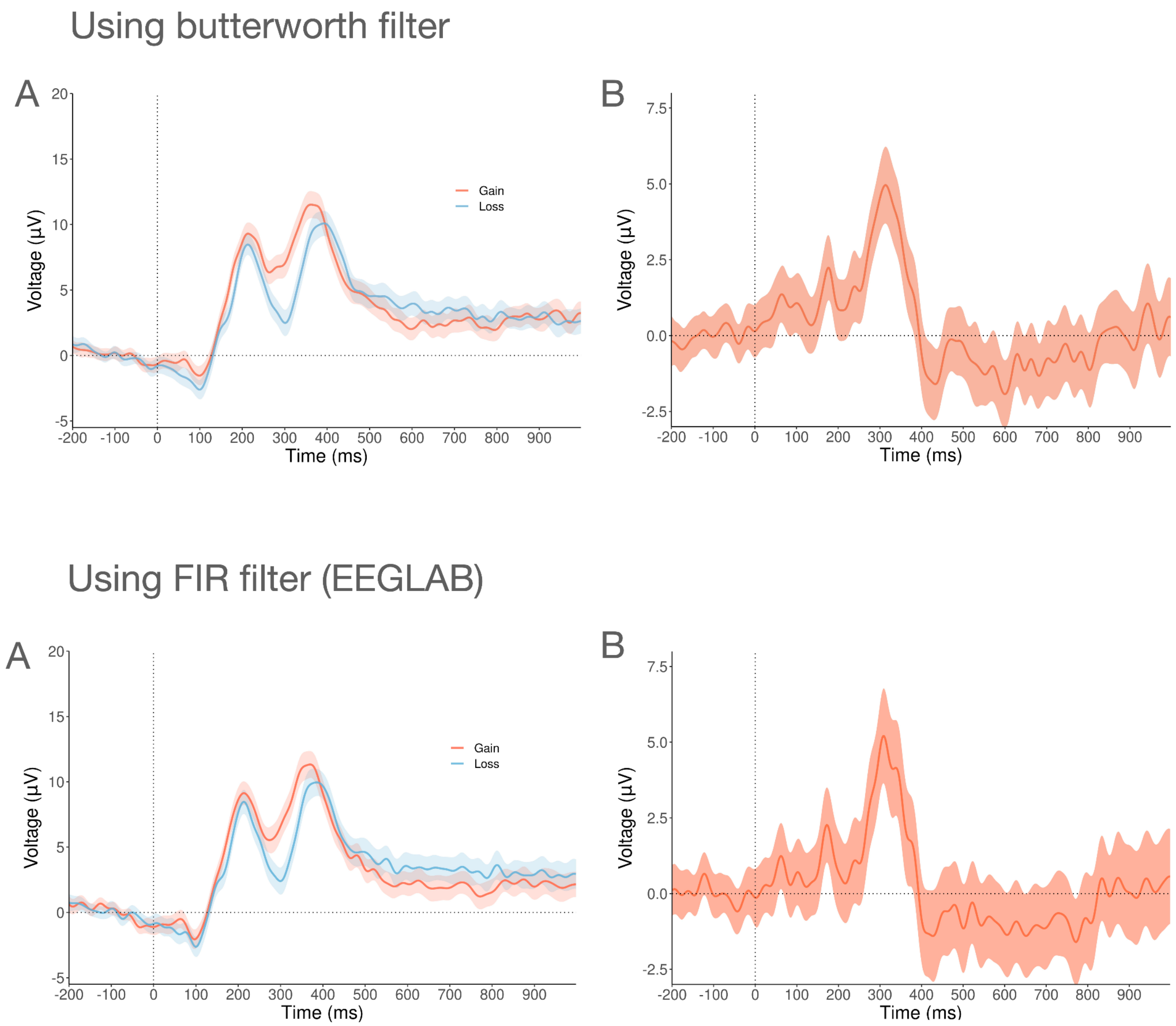

Figure S11. The difference between ERP waveforms after preprocessing by the reference code with the Butterworth filter (as in Williams et al. 2021) and with a FIR filter as designed in EEGLAB, using the automatic way integrated in each tool to detect the flat channels with a trial rejection peak-to-peak threshold of 100 microVolts. (A) grand averaged conditional waveforms (ERP averaged across all subjects) with 95% confidence intervals, (B) grand averaged difference waveform with 95% confidence intervals.

Using butterworth filter

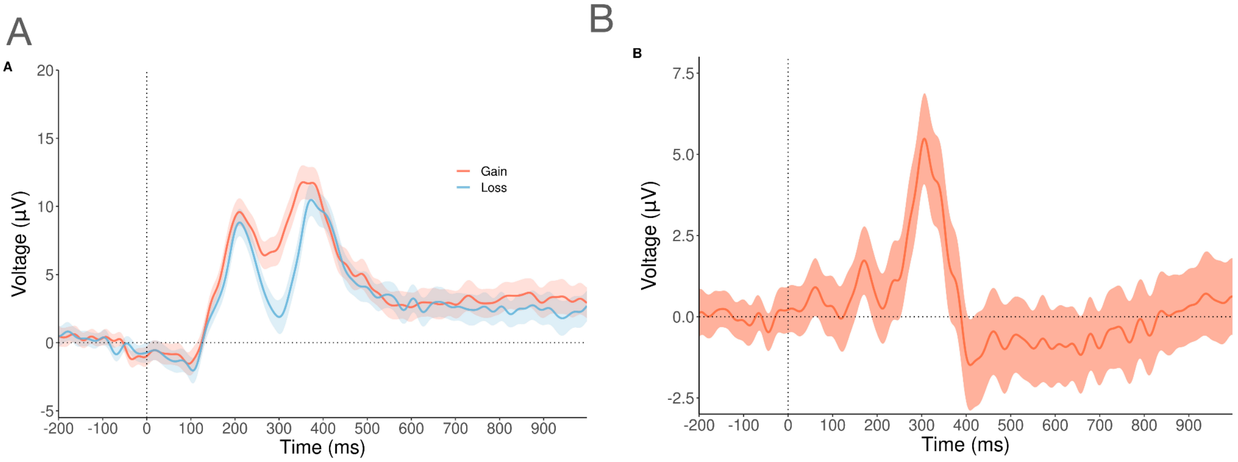

Using FIR filter (EEGLAB)

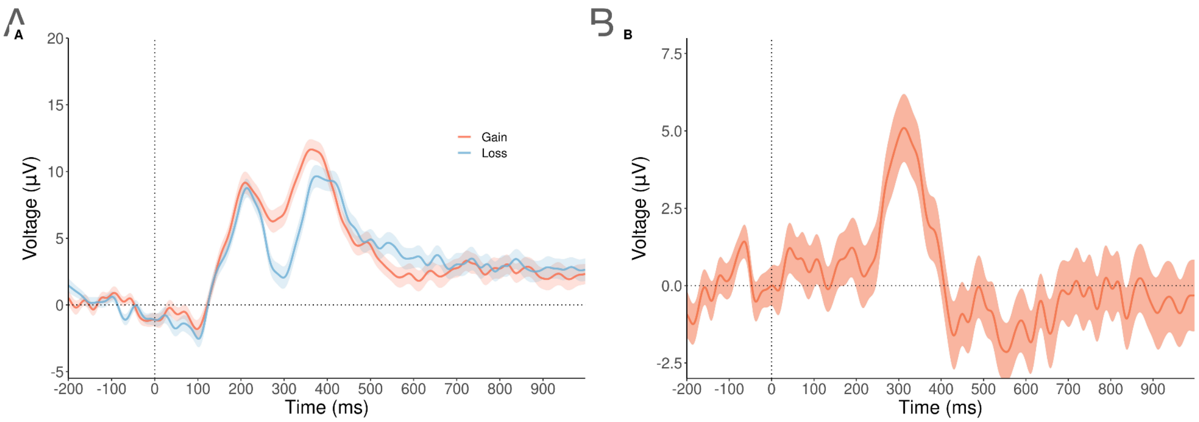

Figure S12. The difference between ERP waveforms after preprocessing by the FieldTrip code with the Butterworth filter (as in Williams et al. 2021) and with a FIR filter as designed in EEGLAB, using the automatic way integrated in each tool to detect the flat channels with a trial rejection peak-to-peak threshold of 100 microVolts. (A) grand averaged conditional waveforms (ERP averaged across all subjects) with 95% confidence intervals, (B) grand averaged difference waveform with 95% confidence intervals.

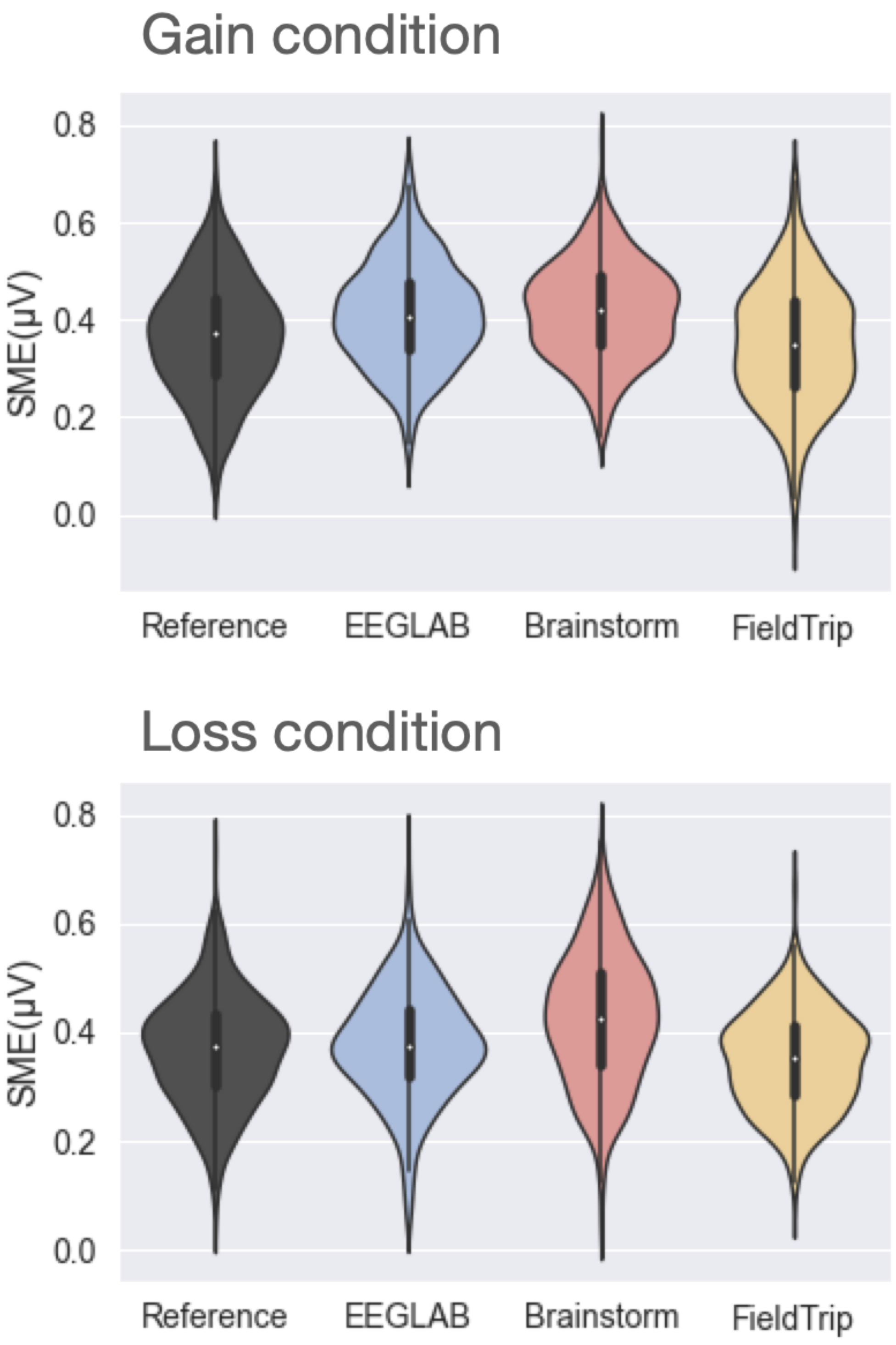

Figure S13. The distributions of the bootstrapping mean peak SME obtained across the 500 subjects by the different software packages, for gain and loss conditions.

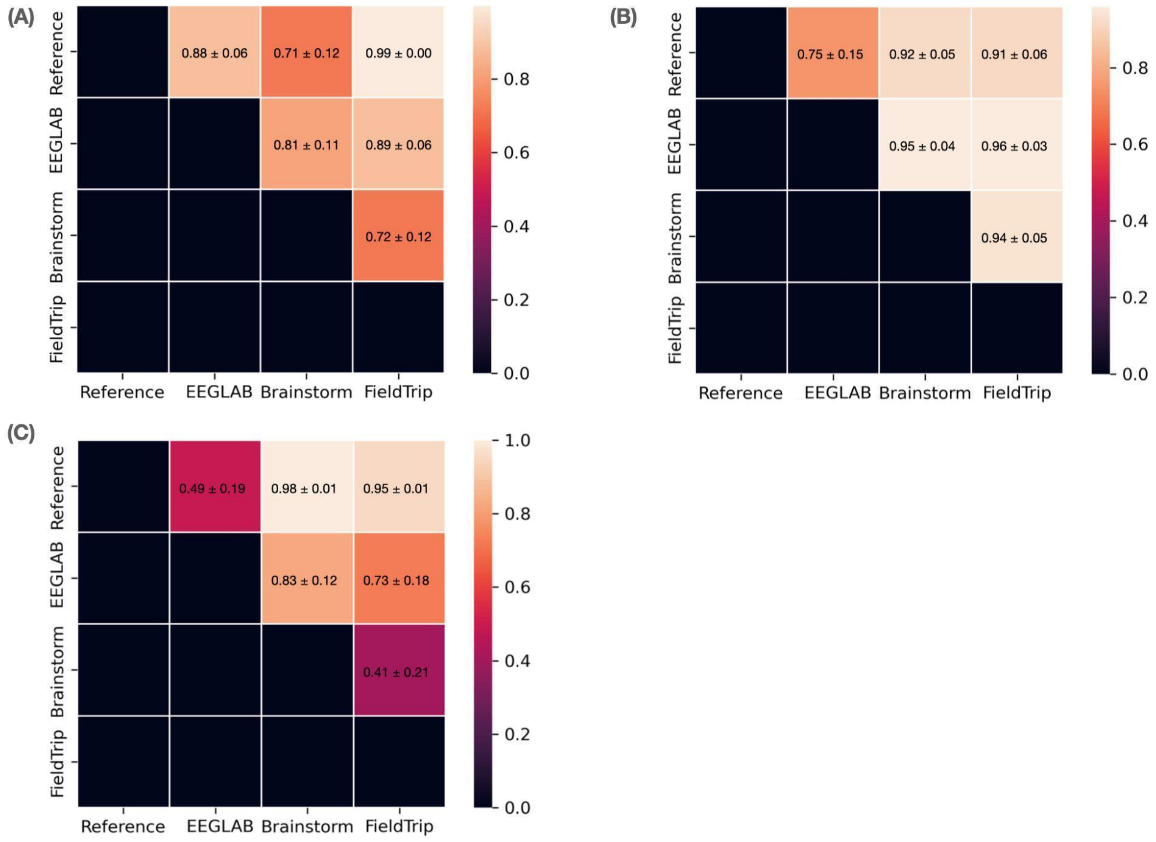

Figure S14. (A) Correlations between the filtered EEG signals obtained by the different filters. (B) Correlations between the trials affiliation obtained by the different trial rejection methods of the software packages. (C) Correlations between the channels affiliation obtained by the different channel rejection methods of the software packages.

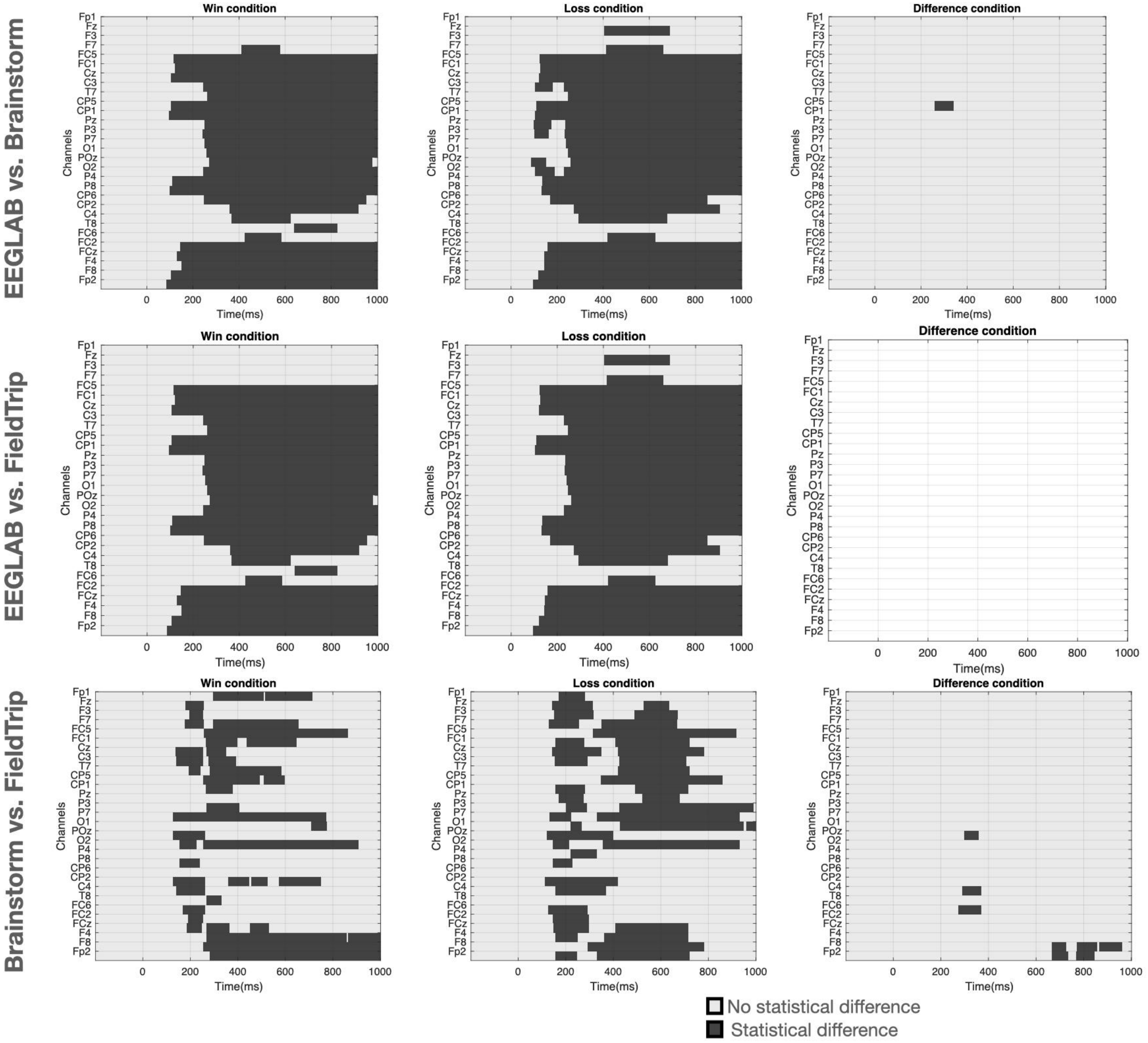

Figure S15. The statistical maps showing the cross-package differences in results at each timepoint and channel using the original pipeline.

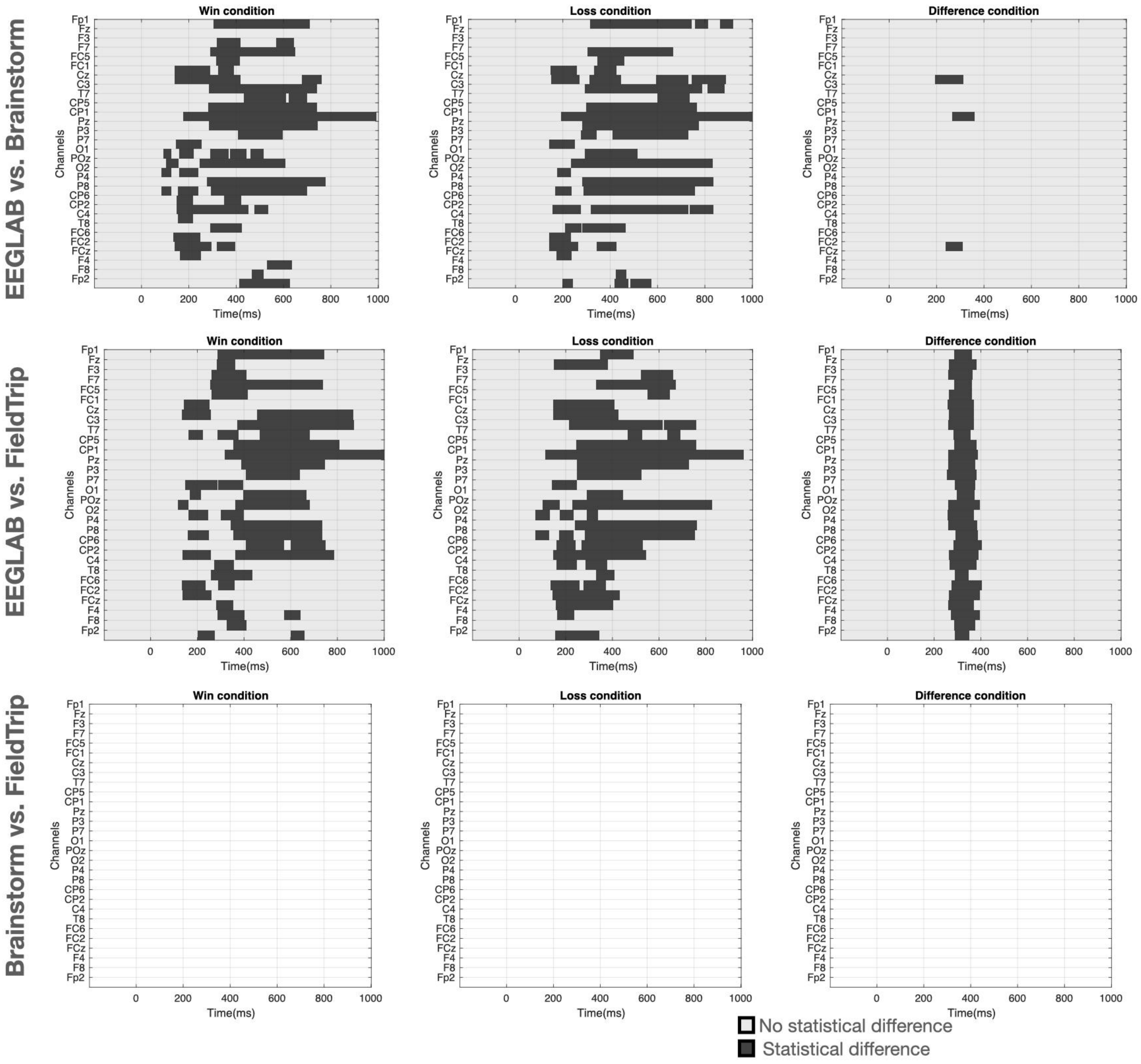

Figure S16. The statistical maps showing the cross-package differences in results at each timepoint and channel using the flat channel detection way with a 100 microVolt peak-to-peak criterion.

		Mean [95% CI]	Standard deviation	Cohen's d
**Original paper (Williams et al. 2021)**	**Mean**	3.70 [3.34 , 4.07]	4.11	0.90 [0.77, 1.03]
	**Maximum**	7.82 [7.42 , 8.23]	4.59	1.71 [1.56, 1.85]
	**Base to peak**	10.52 [10.12 , 10.91]	4.49	2.34 [2.18, 2.50]
**Reference**	**Mean**	3.92 [3.24 , 4.12]	7.64	0.72 [0.68 , 0.91]
	**Maximum**	13.40 [12.80 , 14.10]	10.11	1.88 [1.79 , 2.02]
	**Base to peak**	19.69 [18.80 , 20.30]	12.80	2.16 [1.89 , 2.30]
**EEGLAB**	**Mean**	2.84 [2.54 , 3.13]	8.25	0.48 [0.41 , 0.55]
	**Maximum**	12.94 [12.23 , 13.30]	8.92	2.05 [1.93 , 2.32]
	**Base to peak**	20.2 [19.70 , 21.07]	10.90	2.60 [2.30 , 2.92]
**Brainstorm**	**Mean**	3.84 [3.19 , 4.49]	6.24	0.80 [0.69 , 1.04]
	**Maximum**	9.74 [9.05 ,10.43]	6.66	2.07 [1.81 , 2.32]
	**Base to peak**	13.38 [12.6 ,14.13]	7.22	2.62 [2.31 , 2.93]
**FieldTrip**	**Mean**	3.40 [2.66 , 4.15]	8.46	0.56 [0.43 , 0.70]
	**Maximum**	12.22 [11.38 ,13.00]	9.50	1.81 [1.61 , 2.01]
	**Base to peak**	17.70 [16.79 , 18.79]	11.30	2.22 [1.90 , 2.45]

Table S1, Mean, maximum, and base to peak and the effect size of the reward positivity (the difference ERP) for the reference paper as well as the three studied software packages EEGLAB BrainsStorm and FieldTrip using the automatic way integrated in each tool to detect the flat channels with a trial rejection peak-to-peak threshold of 100 microVolts. The reported mean (in μV) , standard deviation (in μV) and Cohen's d values were computed across subjects. This table is reproduced from Table 1 reported in (Williams et al. 2021).

		Mean [95% CI]	Standard deviation	Cohen's d [95% CI]
**Original paper (Williams et al. 2021)**	**Gain**	8.02 [7.54 , 8.49]	5.38	1.49 [1.35, 1.63]
	**Loss**	4.96 [4.53 , 5.38]	4.87	1.02 [0.89, 1.15]
**Reference**	**Gain**	7.30 [6.60 , 8.30]	6.80	1.50 [1.42 , 1.63]
	**Loss**	3.96 [3.45 , 4.12]	6.80	0.82 [0.71 , 0.93]
**EEGLAB**	**Gain**	4.92 [4.79 , 4.12]	6.67	1.04 [0.95 , 1.18]
	**Loss**	2.58 [2.10 , 2.90]	5.65	0.65 [0.54, 0.77]
**Brainstorm**	**Gain**	8.43 [7.30 , 9.14]	6.80	1.75 [1.52 1.98]
	**Loss**	5.60 [4.98 , 6.34]	6.57	1.21 [1.03 1.41]
**FieldTrip**	**Gain**	7.32 [6.60 , 7.9]	7.53	1.37 [1.20 1.55]
	**Loss**	4.67 [4.08 , 5.26]	6.70	0.98 [0.83 1.13]

Table S2. Effect size of the gain and loss conditional ERP, using the meak peak measure, for all software using the automatic way integrated in each tool to detect the flat channels with a trial rejection peak-to-peak threshold of 100 microVolts. . The reported mean (in µV), standard deviation (in µV) and Cohen's d values were computed across subjects. This table is reproduced from Table 2 reported in (Williams et al. 2021).

		Mean [95% CI]	Standard deviation	Cohen's d [95% CI]
Original paper (Williams et al. 2021)	Mean	3.70	4.11	0.90
	Maximum	7.82	4.59	1.71
	Base to peak	10.52	4.49	2.34
Reference	Mean	3.87	6.70	0.81
	Maximum	10.90	8.04	1.90
	Base to peak	15.42	9.00	2.39
EEGLAB	Mean	3.08	7.27	0.59
	Maximum	11.18	9.04	1.74
	Base to peak	16.94	13.14	1.82
Brainstorm	Mean	2.67	11.00	0.34
	Maximum	13.64	11.20	1.71
	Base to peak	19.90	10.72	2.62
FieldTrip	Mean	4.27	10.50	0.57
	Maximum	15.54	11.50	1.89
	Base to peak	23.47	15.10	2.19

Table S3. Mean, maximum, and base to peak and the effect size of the reward positivity (the difference ERP) for the reference paper as well as the three studied software packages EEGLAB BrainsStorm and FieldTrip using the automatic way integrated in each tool to detect the flat channels with a trial rejection peak-to-peak threshold of 200 microVolts. The reported mean (in μV) , standard deviation (in μV) and Cohen's d values were computed across subjects. This table is reproduced from Table 1 reported in (Williams et al. 2021).

		Mean [95% CI]	Standard deviation	Cohen's d [95% CI]
**Original paper (Williams et al. 2021)**	**Gain**	8.02	5.38	1.49
	**Loss**	4.96	4.87	1.02
**Reference**	**Gain**	5.65	6.67	1.19
	**Loss**	3.00	6.74	0.62
**EEGLAB**	**Gain**	5.74	8.69	0.90
	**Loss**	3.36	7.36	0.64
**Brainstorm**	**Gain**	9.79	8.54	1.62
	**Loss**	7.64	8.87	1.20
**FieldTrip**	**Gain**	7.16	8.14	1.24
	**Loss**	3.57	7.61	0.66

Table S4. Effect size of the gain and loss conditional ERP, using the meak peak measure, for all software using the automatic way integrated in each tool to detect the flat channels with a trial rejection peak-to-peak threshold of 200 microVolts. . The reported mean (in µV), standard deviation (in µV) and cohen's d values were computed across subjects. This table is reproduced from Table 2 reported in (Williams et al. 2021).

Pipeline	Original script	EEGLAB	Brainstorm	FieldTrip	Common subjects/trials
**Paper's channel detection + trial rejection peak-to-peak threshold =100**	N_subjects = 497<br>N_trials = 21906	N_subjects = 453<br>N_trials =17957	N_subjects = 414<br>N_trials= 10678	N_subjects = 441<br>N_trials= 21042	N_subjects=414<br>N_trials= 10665
**Flat channels detection + trial rejection peak-to-peak threshold =100**	N_subjects = 264<br>N_trials = 6706	N_subjects = 191<br>N_trials= 4208	N_subjects =213<br>N_trials = 4005	N_subjects = 397<br>N_trials = 10213	N_subjects = 191<br>N_trials= 4000
**Flat channels detection) + trial rejection peak-to-peak threshold =200**	N_subjects = 416<br>N_trials = 22164	N_subjects = 404<br>N_trials = 15414	N_subjects = 365<br>N_trials = 16032	N_subjects = 482<br>N_trials = 15489	N_subjects = 365<br>N_trials = 15414

Table S5. The number of subjects and trials kept after adapting the different proposed pipelines.

The last column shows the number of common trials/subjects kept between all tools.

Exploring the independent effect of the filtering, the trial rejection and the channel detection methods:

Owing to the fact that assessing the effect induced by each processing step will help to better understand the possible reasons of the observed discrepancy between tools, we studied the independent effect of 1- the filtering method, 2- the trial rejection method, 3- the channel detection method on the results. To explore the effect of each factor, the same data were set as inputs for the different methods, and the immediate results were statistically compared.

1- Effect of the filtering methods: The raw signals of the 500 participants were filtered using the four filters : the Butterworth filter used in the reference paper, the FIR filter designed in EEGLAB, the FIR filter designed in Brainstorm, and the Butterworth filter designed in FieldTrip. Then, for each participant, we assessed the correlation between the filtered signals derived between each two tools (Pearson's correlation). Figure S14. A shows that the highest correlation is reached between the reference and FieldTrip filters, and the lowest correlation is obtained between Brainstorm and the reference filters.

2- Effect of the trial rejection methods: The filtered signals generated by the reference filter were segmented into epochs and then bad epochs were detected using the different tools. Then, for each participant, we assessed the correlation between the trials affiliation (bad or good) using the Tanimoto metric (defined as the ratio of the

intersection of the two compared sets over the union of the two sets). Figure S14.B shows that the lowest correlation is observed between the reference and EEGLAB, while high correlations (>0.9) are reached between the reference and the other tools. It is noteworthy to mention here that EEGLAB does not reject trials based on a peak-to-peak voltage threshold but rather on both the minimum and the maximum thresholds. This criterion might reject trials that are exceeding the maximum or minimum thresholds but not necessarily exceeding the peak-to-peak threshold.

3- Effect of the channel detection methods: The difference between the channel affiliation (bad or good) detected by the different tools is assessed using Tanimoto. Figure S14.C shows that the lowest correlation is obtained between the reference and EEGLAB results. These results are in concordance with the correlations obtained in Figure S14.B, as the channel detection method is dependent on the trial rejection rate.
